# Non-uniformity of Changes in Drug-Metabolizing Enzymes and Transporters in Liver Cirrhosis: Implications for Drug Dosage Adjustment

**DOI:** 10.1021/acs.molpharmaceut.1c00462

**Published:** 2021-08-24

**Authors:** Eman El-Khateeb, Brahim Achour, Zubida M. Al-Majdoub, Jill Barber, Amin Rostami-Hodjegan

**Affiliations:** † Centre for Applied Pharmacokinetic Research, University of Manchester, Manchester M13 9PT, U.K.; ‡ Clinical Pharmacy Department, Faculty of Pharmacy, Tanta University, Tanta 31527, Egypt; § Certara UK Ltd. (Simcyp Division), Sheffield S1 2BJ, U.K.

**Keywords:** cirrhosis, hepatic impairment, quantitative proteomics, enzymes, transporters, PBPK

## Abstract

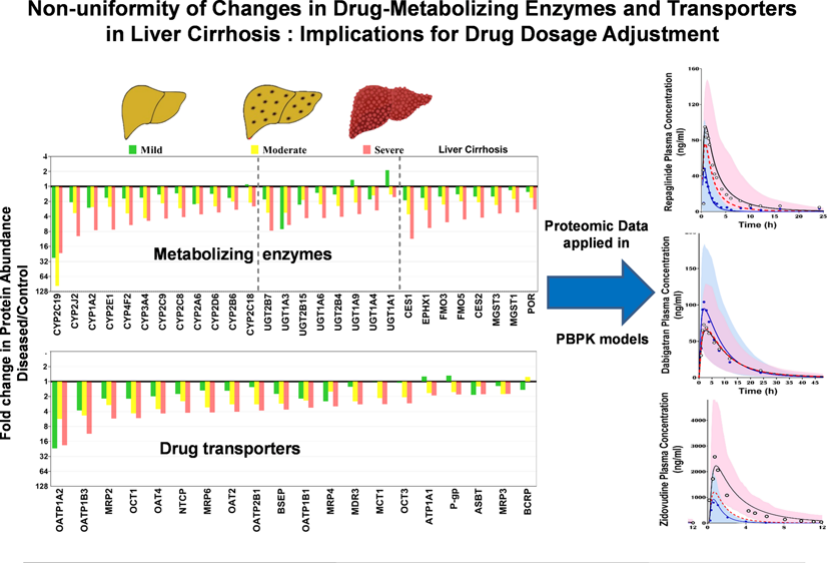

Liver cirrhosis is a chronic disease that affects the liver structure, protein expression, and overall metabolic function. Abundance data for drug-metabolizing enzymes and transporters (DMET) across all stages of disease severity are scarce. Levels of these proteins are crucial for the accurate prediction of drug clearance in hepatically impaired patients using physiologically based pharmacokinetic (PBPK) models, which can be used to guide the selection of more precise dosing. This study aimed to experimentally quantify these proteins in human liver samples and assess how they can impact the predictive performance of the PBPK models. We determined the absolute abundance of 51 DMET proteins in human liver microsomes across the three degrees of cirrhosis severity (*n* = 32; 6 mild, 13 moderate, and 13 severe), compared to histologically normal controls (*n* = 14), using QconCAT-based targeted proteomics. The results revealed a significant but non-uniform reduction in the abundance of enzymes and transporters, from control, by 30–50% in mild, 40–70% in moderate, and 50–90% in severe cirrhosis groups. Cancer and/or non-alcoholic fatty liver disease-related cirrhosis showed larger deterioration in levels of CYP3A4, 2C8, 2E1, 1A6, UGT2B4/7, CES1, FMO3/5, EPHX1, MGST1/3, BSEP, and OATP2B1 than the cholestasis set. Drug-specific pathways together with non-uniform changes of abundance across the enzymes and transporters under various degrees of cirrhosis necessitate the use of PBPK models. As case examples, such models for repaglinide, dabigatran, and zidovudine were successful in recovering disease-related alterations in drug exposure. In conclusion, the current study provides the biological rationale behind the absence of a single dose adjustment formula for all drugs in cirrhosis and demonstrates the utility of proteomics-informed PBPK modeling for drug-specific dose adjustment in liver cirrhosis.

## Introduction

Cirrhosis is a global health burden, accounting for over 1 million deaths per annum, and 4.9 to 9.5% of the global population is believed to have some level of cirrhosis.^1−3^ It occurs in late-stage liver fibrosis as a result of different types of liver disease, such as hepatitis, cholestasis, cancer, and alcoholic and non-alcoholic fatty liver disease (NAFLD).^4^ It leads to alterations to hepatic architecture, which cause changes in blood flow, protein binding, and expression of drug-metabolizing enzymes (DMEs).^5^ These changes lead to variable pharmacokinetics (PK) of many drugs in cirrhotic populations, compared with healthy subjects, through multiple mechanisms, such as a reduction in the absolute number of functioning cells in the liver, changes in abundance and/or activity of enzymes in surviving hepatocytes, and impaired drug and oxygen entry into liver cells.^6^ This leads to a decrease in the liver’s capacity to eliminate drugs and may require specific drug dosage adjustment.^7^

In drug development, dedicated PK studies on patients with different degrees of hepatic impairment (HI) are recommended; however, such studies are not conducted for most drugs approved by regulatory agencies, and patients with HI currently receive these drugs with no dosage guidance.^8^ Recent FDA guidelines recommended the inclusion of HI patients into the early phases of clinical studies with close monitoring of side effects.^9^ Implementation of this recommendation may require time, and alternate approaches, such as the use of physiologically based PK (PBPK) models, are therefore applied for predicting changes in drug exposure and guiding dose adjustment in HI populations.^10^ These HI PBPK models incorporate either *in vitro* abundance data from immunoblotting studies or *in vivo* activity data using selective probe substrates administered to patients with liver disease.^5^ The highlighted strategies are limited to protein targets that have specific antibodies or probe substrates. More recently, the use of LC–MS proteomics in the quantification of phase I and II enzymes as well as transporters has contributed useful data, which have so far been limited to only the severe stage of cirrhosis and do not cover some key etiologies of the disease.^11,12^ Therefore, the aim of this study was to assess the impact of cirrhosis at different degrees of disease severity, classified according to the Child–Pugh (CP) system^13^ (as mild, CP-A; moderate, CP-B; and severe, CP-C). Furthermore, the possible effects of disease etiologies associated with cirrhosis, such as NAFLD, alcoholic fatty liver, biliary disease, and cancer, on the expression of enzymes and transporters were investigated.

## Materials and Methods

### Liver Samples and Donor Characteristics

Human liver microsomal (HLM) samples (*n* = 46) representing four sets, that is, the control group in which liver samples were excised from histologically normal areas adjacent to tumors (*n* = 14, Table S1) and three cirrhotic groups (*n* = 32, Table S2), divided according to the severity of cirrhosis using CP scoring into CP-A or mild cirrhosis group (*n* = 6), CP-B or moderate cirrhosis group (*n* = 13), and CP-C or severe cirrhosis group (*n* = 13). These 32 cirrhosis samples were also subdivided according to the liver disease associated with cirrhosis into NAFLD (*n* = 8), biliary disease (*n* = 13), cancer (*n* = 9), and alcoholic fatty liver disease (*n* = 2).

Individual liver tissue samples were provided by Cambridge University Hospitals Tissue Bank (Cambridge, UK), and HLM fractions were prepared by differential centrifugation, as reported previously.^14^ This study is covered by ethical approval from the Health Research Authority and Health and Care Research Wales (HCRW) (Research Ethics Committee Approval Reference 18/LO/1969). Anonymized demographic and clinical data for the donors were previously reported^14^ and are summarized in Tables S1 and S2. The average age for the control group was 66 years (range: 36–83 years). The average age of cirrhosis patients was 60 years (range: 39–70 years). The percentage of female subjects was 29% in the control group and 39% in the cirrhosis group. In addition to individual samples, a pool of normal samples was prepared by mixing 6 μL from each individual HLM fraction and was used to assess the analytical variability between and within batches of samples.

### Sample Preparation for Proteomics

Three concatenated concatemers (QconCATs) were spiked into 70 μg of each individual HLM sample as internal standards: 0.351 μg of MetCAT [QconCAT standard for the quantification of cytochrome P450 enzymes (CYPs) and uridine-5′-diphospho-glucuronosyltransferases (UGTs)], 0.450 μg of NuncCAT [QconCAT for the quantification of non-CYP, non-UGT enzymes], and 0.165 μg of TransCAT [QconCAT for the quantification of transporters]. The samples were also spiked with a mixture of unlabeled exogenous protein standards [0.126 μg of bovine serum albumin, 0.037 μg of yeast aldehyde dehydrogenase (ADH), and 0.168 μg of horse myoglobin] to monitor experimental conditions and enable label-free quantification of the liver proteome.

Filter-aided sample preparation^15^ was used for sample preparation, as previously described with minor modifications.^16,17^ Sample mixtures were solubilized by incubation with sodium deoxycholate (10% w/v final volume), 1,4-dithiothreitol was added at a final concentration of 100 mM, and the protein mixture was incubated at room temperature for 10 min. Reduction of protein disulfide bonds was carried out by incubation at 56 °C for 30 min. Amicon Ultra 0.5 mL centrifugal filters, 10 kDa molecular weight cut-off, (Millipore, Nottingham, UK) were conditioned by brief centrifugation of 400 μL of 0.1 M Tris-HCl, pH 8.5, at 14,000*g* at room temperature. Protein samples were then transferred to the conditioned filter units, followed by centrifugation at 14,000*g* at room temperature for 30 min. Alkylation of reduced cysteine residues was performed by incubation with 100 μL of 50 mM iodoacetamide in the dark for 30 min at room temperature. After alkylation, deoxycholate removal was performed by buffer exchange using two successive washes with 8 M urea in 100 mM Tris-HCl (pH 8.5), 200 μL each. To reduce urea concentration, additional washes (3 × 200 μL) were performed using 1 M urea in 50 mM ammonium bicarbonate (pH 8.5). For each wash, the buffer (200 μL) was added to the filter, without mixing, and centrifuged at 14,000*g* at room temperature for 20 min, leaving a volume of approximately 20 μL in the filter. The filtrate, containing small molecules such as detergent, was discarded. Protein digestion was achieved using lysyl endopeptidase (Lys-C) twice (Lys-C: protein ratio 1:50, 2 h each, at 30 °C), then trypsin digestion was carried out (trypsin: protein ratio 1:25) for 12 h at 37 °C, and another equivalent treatment for an extra 6 h incubation. Peptides were recovered from the filter by centrifugation (14,000*g*, 20 min); a second collection was achieved by adding 0.5 M NaCl (100 μL) to the filter and centrifugation at 14,000*g* for another 20 min. The collected peptides were lyophilized to dryness using a vacuum concentrator at 30 °C and with vacuum in the aqueous mode; the time required was in the range 1–3 h and was sample-dependent. The lyophilized peptides were reconstituted in 20% (v/v) acetonitrile in water, acidified with 2% (v/v) trifluoroacetic acid, and then desalted using C18 spin columns according to the manufacturer’s instructions (Nest Group, USA). The peptides were lyophilized and stored at −80 °C until mass spectrometric analysis.

### Liquid Chromatography with Tandem Mass Spectrometry

Lyophilized peptides were resuspended in 70 μL of 3% (v/v) acetonitrile in water with 0.1% (v/v) formic acid. Digested samples were analyzed by liquid chromatography with tandem mass spectrometry (LC–MS/MS) using an UltiMate 3000 Rapid Separation LC (RSLC, Dionex Corporation, Sunnyvale, CA) coupled to a Q Exactive HF Hybrid Quadrupole-Orbitrap mass spectrometer (Thermo Fisher Scientific, Waltham, MA). Mobile phase A was 0.1% formic acid in water and mobile phase B was 0.1% formic acid in acetonitrile, and peptides were eluted on a CSH C18 analytical column (75 mm × 250 μm inner diameter, 1.7 μm particle size) (Waters, UK). A 1 μL aliquot of the sample was transferred to a 5 μL loop and loaded onto the column at a flow rate of 300 nL/min for 5 min at 5% B. The loop was then taken out of line, and the flow was reduced from 300 to 200 nL/min over 0.5 min. Peptides were separated using a gradient from 5 to 18% B in 63.5 min, then from 18 to 27% B in 8 min, and finally from 27% B to 60% B in 1 min. The column was washed at 60% B for 3 min before re-equilibration to 5% B in 1 min. At 85 min, the flow was increased to 300 nL/min until the end of the run at 90 min. Mass spectrometry data were acquired in a data-dependent manner for 90 min in the positive mode. Peptides were selected for fragmentation automatically by data-dependent analysis on the basis of the top 12 peptides with *m*/*z* between 300 and 1750 Th and a charge state of 2+, 3+, and 4+ with a dynamic exclusion set at 15 s. The MS resolution was set at 120,000 with an AGC target of 3E6 and a maximum fill time set at 20 ms. The MS2 resolution was set to 30,000, with an AGC target of 2E5, a maximum fill time of 45 ms, an isolation window of 1.3 Th, and a collision energy of 28 eV.

### Proteomic Data Analysis

Proteins were identified by searching peptide MS/MS data against the UniProtKB database (http://www.uniprot.org/) using MaxQuant version 1.6.10.43 (Max Planck Institute of Biochemistry, Martinsried, Germany). QconCAT-based quantification was carried out as previously described^18−20^ to measure 15 CYPs and 9 UGTs (MetCAT), in addition to UGT2B17, 22 non-CYP/non-UGT DMEs (NuncCAT), and 30 transporters (TransCAT). A protein was considered quantifiable in liver microsomal samples if (a) there was evidence of its expression in the liver (Human Protein Atlas, https://www.proteinatlas.org/), (b) it was localized in a membrane (Uniprot, https://www.uniprot.org/), (c) it was identified by at least one razor or one unique peptide, and (d) it was detected in a sufficient number of samples (at least 3 samples/group). A list of the peptides that constitute the QconCATs used in this study is presented in Table S3. The abundance of each target protein was calculated using eq 1.
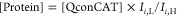
1where [Protein] is the protein abundance based on the surrogate peptide *i*, measured in units of pmol/mg microsomal protein. *I*_*i*,L_/*I*_*i*,H_ is the ratio of the intensity of the light (analyte) to the heavy (QconCAT-derived) surrogate peptide, and [QconCAT] is the concentration of the QconCAT standard measured using eq 2.
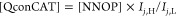
2where *I*_*j*,H_/*I*_*j*,L_ is the ratio of the intensity of the heavy (QconCAT-derived) to the light (spiked in) NNOP standard peptide, and [NNOP] is the concentration of the NNOP peptide standard expressed in units of pmol/mg microsomal protein analyzed by the mass spectrometer. The intensity ratios were corrected for isotope labeling efficiency prior to use in the equations.^21,22^ Unlabeled NNOP peptides, EGVNDNEEGFFSAR, GVNDNEEGFFSAR, and AEGVNDNEEGFFSAR, were added for every 70 μg of starting protein samples at 376, 700, and 156 fmol, respectively, to quantify the TransCAT, MetCAT, and NuncCAT, respectively. The three QconCATs have shown comparable results to label-free quantification methods in a previous study.^23^

The measured abundance values of each protein were scaled up to their corresponding levels in tissue (pmol/g liver) using individual microsomal protein content per gram of liver (MPPGL) for each sample. The preparation of the microsomal fraction and details on the determination of MPPGL for the same set of samples is explained in detail in our previous publication.^14^ Briefly, the microsomes were separated by sequential ultracentrifugation and the protein content in the resulting fraction was measured using bicinchoninic acid assay and corrected for the microsomal protein loss using the activity of a specific marker enzyme namely cytochrome P450 reductase.^14^

### Assessment of the Degree of Technical and Analytical Variability

Nine samples, representing all disease groups (2 normal, 2 cancer, 1 alcohol, 2 cholestasis, and 2 NAFLD samples), were prepared in triplicate and analyzed by LC–MS/MS under the same conditions. The data were used to assess technical variability in quantification. A pool of normal samples (*n* = 14) was prepared once and analyzed twice in each of 5 batches of samples (10 overall runs) to assess intra- and inter-batch variability. Technical and batch-to-batch variability was evaluated using the coefficient of variation of replicates from different analyses in each set and across batches.

### Comparing Abundance of Enzymes and Transporters Among Disease Groups

The absolute abundance values of the quantified liver enzymes and transporters in cirrhotic livers (classified either according to CP score or according to the disease etiology or associated liver disease) were compared and assessed against abundance in the control group. Targets that were detected in at least 3 samples per group were included in the comparison; therefore, the alcohol-related cirrhosis group (2 samples) was excluded from the etiology comparison. To rule out the confounding effect of disease severity, this comparison was restricted to moderate disease, which was the only disease grade that included a sufficient number of samples in each etiology.

### Statistical Analysis

The samples were classified based on disease severity, using the CP score, and according to the associated disease. Statistical analysis of the data was carried out and graphs were created using GraphPad Prism version 7.0 (La Jolla, California, USA). Shapiro–Wilk normality test was applied to assess the normality of the distribution of the data. In the absence of normal distribution, non-parametric statistics was used, and the data were presented as median and 95% confidence interval (CI). Equality of variance was assessed by a modified Levene’s test (Brown–Forsythe test). The abundance data from different groups were not normally distributed (Shapiro–Wilk test, *p* < 0.05), and variance within severity groups was homogeneous (Brown–Forsythe test, *p* > 0.05). Accordingly, non-parametric statistics was used to assess differences between groups (Kruskal–Wallis and *post-hoc* Mann–Whitney tests) with statistical significance cut-off set at 0.05. Similar ANOVA analysis with *post-hoc* tests was used to compare the data for the control group and three disease etiologies (cancer, cholestasis, and NAFLD) at the same degree of severity of cirrhosis (moderate set). Statistical significance was again considered with a cut-off *P*-value of 0.05 and Bonferroni-corrected for multiple iterations to *p* < 0.0085* and *p* < 0.0017** (six iterations). Correlation between the abundance of hepatic UGTs or transporters and log-transformed total serum bilirubin for each individual patient was performed using the Spearman test (*R*_s_) as transporter and UGT abundances were not normally distributed (Shapiro–Wilk test, *p* < 0.05). Linear regression was used to assess the scatter of the data. Correlations were considered significant if *R*_s_ was at least 0.5 and the probability was <0.05. *R*^2^ between 0.3 and 0.7 was considered a moderate relationship, and >0.7 was considered strong.

### Application of Proteomic Data in PBPK Models of Cirrhosis

Three previously verified (tested against studies not used for building the model) PBPK models were used to confirm the applicability of the collected proteomic data in the prediction of drug exposure in cirrhosis populations. In this modeling exercise, we used repaglinide (an antidiabetic agent and a substrate for CYP2C8, CYP3A4, and OATP1B1), dabigatran etexilate (a prodrug converted by carboxylesterases CES1 and CES2 to the active anticoagulant dabigatran, which is mainly excreted unchanged in urine), and zidovudine (an antiretroviral drug and a substrate for UGT2B7 and, to a lesser degree, metabolized by CYP reductase).

For each drug, simulations with virtual cirrhosis populations were performed using the following two methods and the outputs were compared:

1 Proteomic_sim_cirrhosis method: The disease-to-normal abundance ratio from the current study was used as a scalar for the intrinsic clearance in each cirrhosis population (CP-A, CP-B, or CP-C). As this ratio was based on enzyme abundance per gram of tissue, changes in MPPGL between diseased samples and normal livers have already been accounted for in this ratio. Therefore, the functional liver volume hypothesized by Johnson *et al.*(5) was returned back to normal values measured in healthy populations. Physiological changes other than enzyme abundance per g of liver tissue and liver size scalar were kept the same as those in the population library in Simcyp simulator version 19 (Sheffield, UK), as previously reported by Johnson *et al.*(5) and summarized here in Table S4.
2 Simcyp_cirrhosis method: Default Simcyp V 19 settings in cirrhosis populations were kept the same including abundance data and liver volume scalars presented in Table S4.

Demographic data for both healthy and cirrhosis individuals were reported previously for repaglinide,^24^ dabigatran etexilate,^25^ and zidovudine^26^ and are summarized in Table S5. Drug-specific input parameters and changes in the intrinsic clearance of the three drugs in cirrhosis populations are presented in Table 1. All parameters were derived from the simulator’s library unless otherwise stated, as shown in Table 1. Simulation trials were set to 10 trials of 10 individuals each. The ratio of the area under the curve predicted by the model (AUC_pred_) and the observed value (AUC_obs_) was calculated and considered acceptable if the value was between 0.5- and 2-fold. The ability of the model to predict changes in exposure due to cirrhosis was performed by comparing the ratio of AUC for the diseased population to that for the healthy population (AUCR) in both simulated and observed data. The model’s prediction was considered acceptable if the ratio of predicted AUCR to observed AUCR was between 0.5- and 2-fold.

**Table 1:**
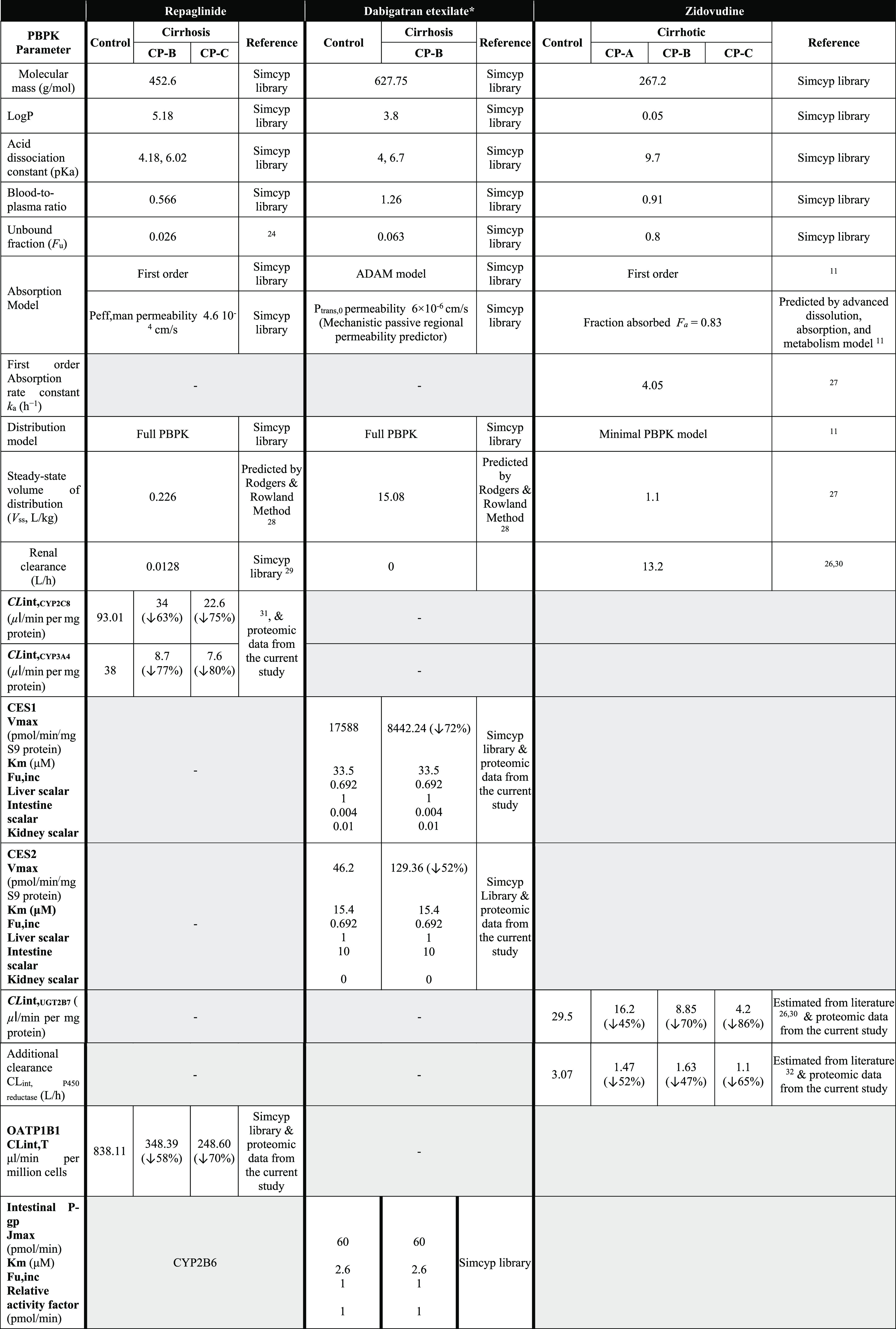
Input Parameters Used for PBPK Simulations of Repaglinide, Dabigatran Etexilate, and Zidovudine

a Dabigatran is the active metabolite that is mainly eliminated by the kidney; the input parameters were kept the same as the default in the Simcyp V19 library as no abundance data were required to be modified.

## Results

### Quality and Scaling of the Proteomic Data

QconCAT-based targeted proteomics was used to determine changes in the protein expression of liver enzymes and transporters across three stages of cirrhosis severity relative to the histologically normal liver. The targets included 14 CYPs, 9 UGTs, 8 non-CYP and non-UGT enzymes, 19 transporters, and 1 membrane marker. Technical and batch-to-batch variabilities were within 30% for 96 and 97% of targets, respectively (Figure S1). The targets that reflected the highest variability (>30%) were not detected consistently (FMO5, MGST3, MRP2, and MDR3). The lower limit of quantification for consistently quantified targets was 0.08 pmol/mg protein (translating to an average tissue content of ∼2 pmol/g liver) based on a cut-off technical variability of 20% in quality control samples.

The abundance levels measured in pmol/mg membrane protein were scaled up to tissue levels using MPPGL values for each individual sample. Individual MPPGL values were previously reported for the same set of samples^14^ and are summarized in Table S6. The median (range) MPPGL for the control group was 37.3 (30.4–63.6 mg/g), whereas for the cirrhotic samples, it was 30.8 (12.9–49.1 mg/g). The measured tissue levels of enzymes and transporters were used in the comparisons among all samples.

### Abundance of DMEs and Transporters in Livers with Different Severities of Cirrhosis

**Figure 1:**
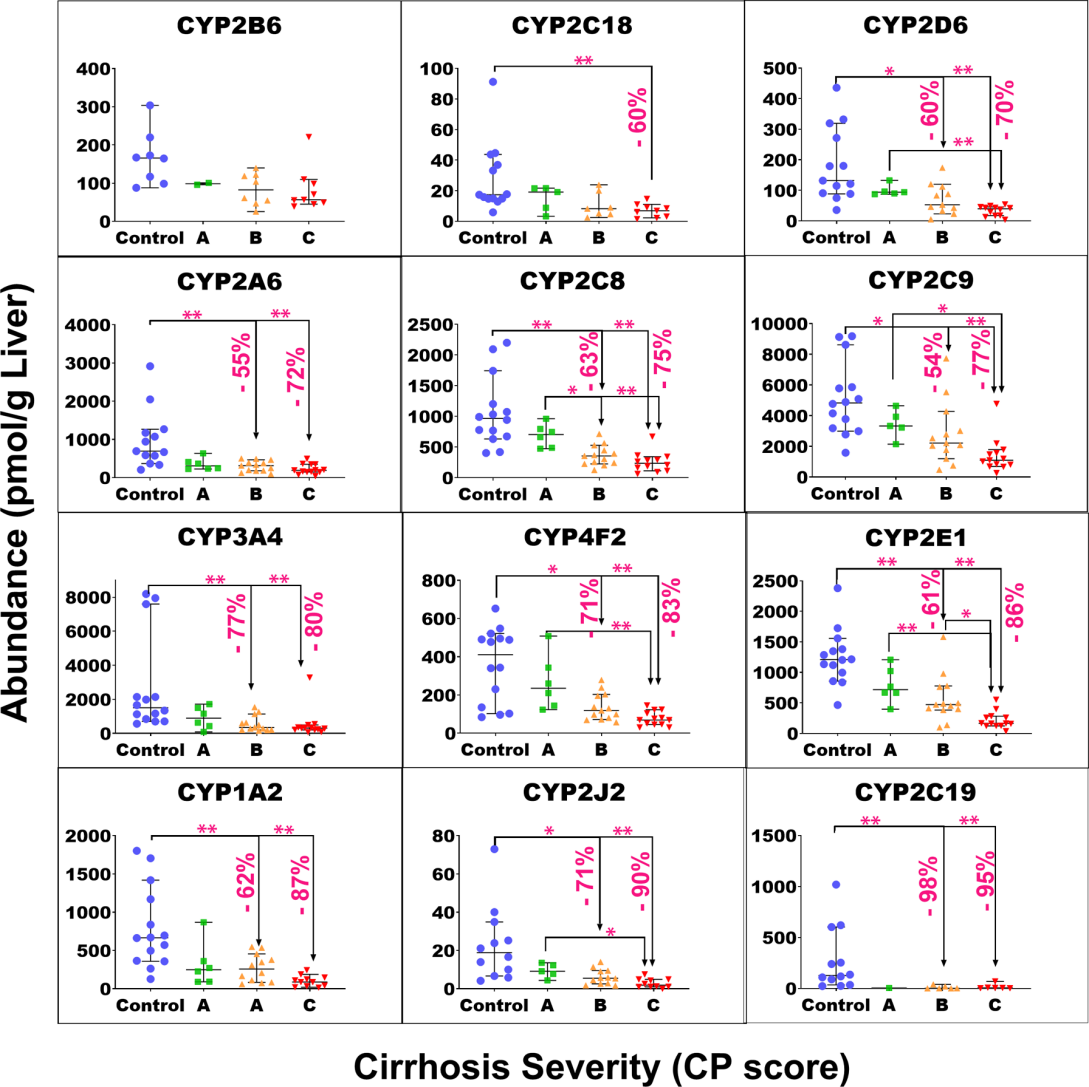
Individual abundance values of cytochrome P450 enzymes in pmol per g of liver tissue from normal control compared to different grades of liver cirrhosis stratified using the Child–Pugh (CP) score [(A) CP-A or mild; (B) CP-B or moderate, and (C) CP-C or severe]. Horizontal lines represent medians, and error bars are the 95% CIs. Stars represent comparisons with statistical significance (*p < 0.0085 and **p < 0.0017), while the percentages represent the degree of change from normal control.

**Figure 2:**
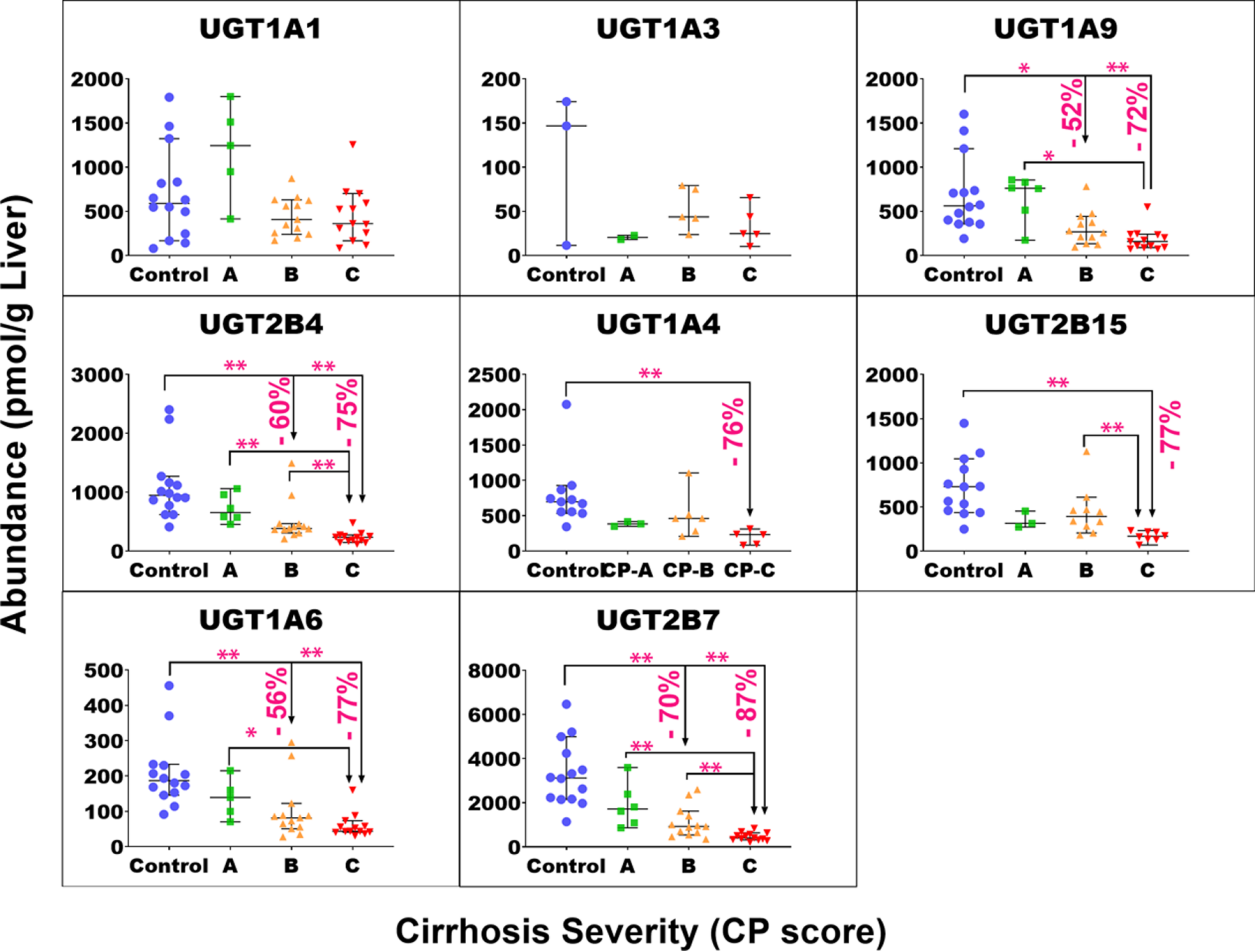
Individual abundance values of UGT enzymes in pmol per g of liver tissue from normal control compared to different grades of liver cirrhosis stratified using the CP score [(A) CP-A or mild; (B) CP-B or moderate, and (C) CP-C or severe]. Horizontal lines represent medians, and error bars are the 95% CIs. Stars represent comparisons that are statistically significant (*p < 0.0085 and **p < 0.0017), while the percentages represent the degree of change from normal control.

**Figure 3:**
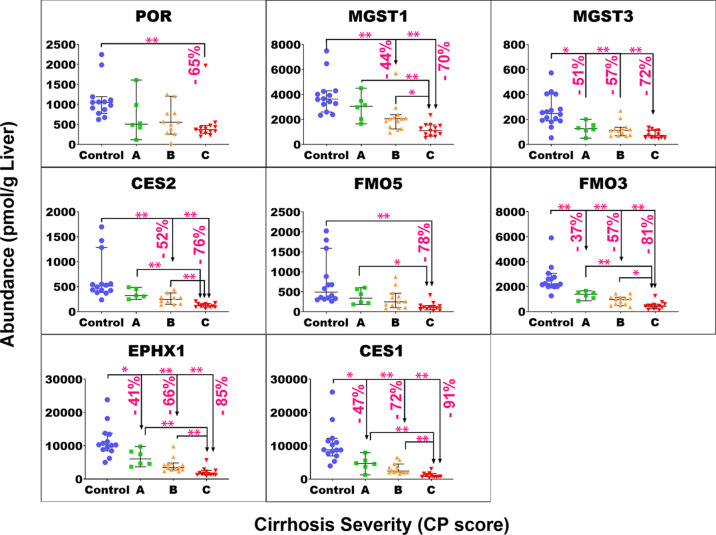
Individual abundance values of non-CYP and non-UGT enzymes in pmol per g of liver tissue from normal control compared to different grades of liver cirrhosis stratified using the CP score [(A) CP-A or mild; (B) CP-B or moderate, and (C) CP-C or severe]. Horizontal lines represent medians, and error bars are the 95% CIs. Stars represent comparisons that are statistically significant (*p < 0.0085 and **p < 0.0017), while the percentages represent the degree of change from normal control.

**Figure 4:**
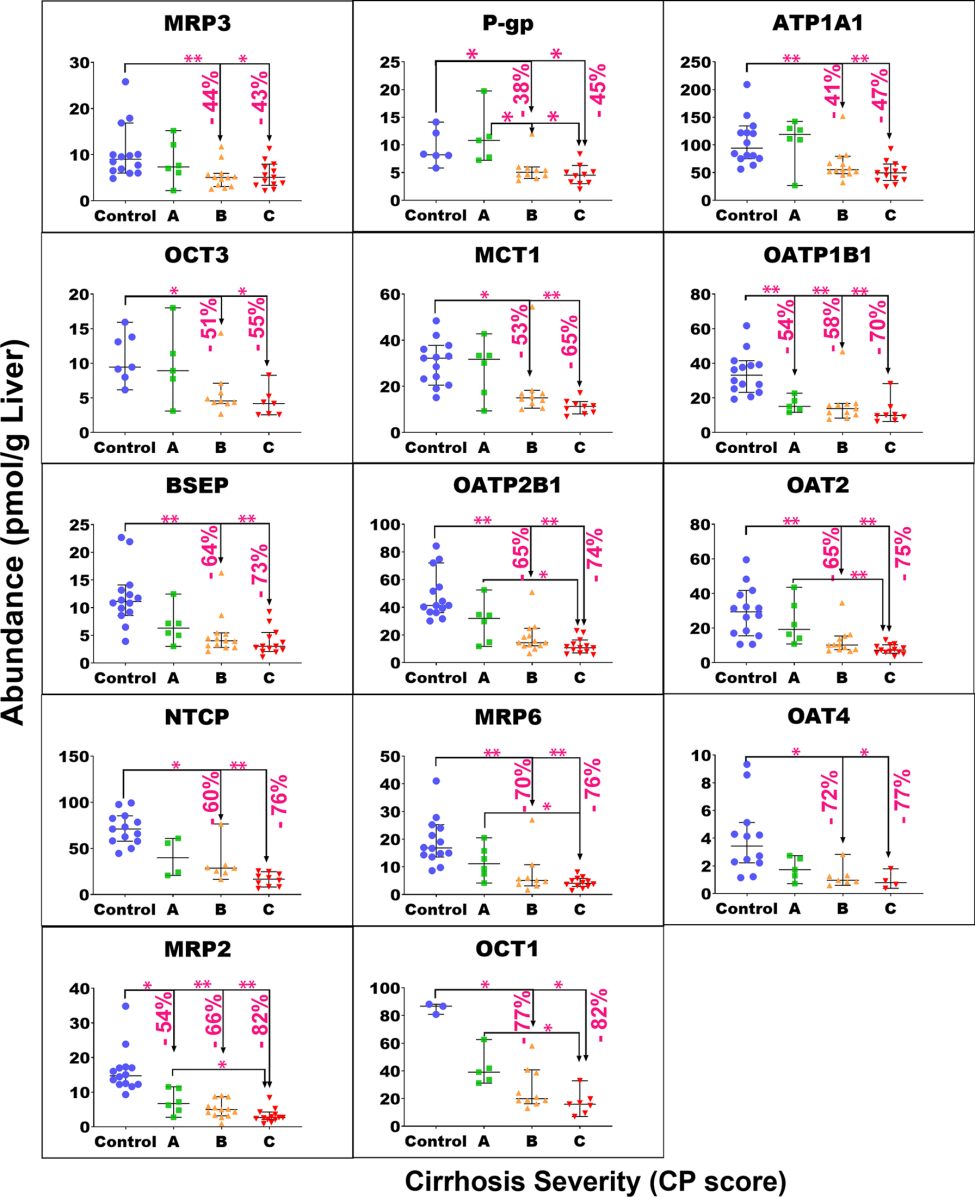
Individual abundance values of transporters in pmol per g of liver tissue from normal control compared to different grades of liver cirrhosis stratified using the CP score [(A) CP-A or mild; (B) CP-B or moderate, and (C) CP-C or severe]. Horizontal lines represent medians, and error bars are the 95% CIs. Stars represent comparisons that are statistically significant (*p < 0.0085 and **p < 0.0017), while the percentages represent the degree of change from normal control.

To assess the effect of cirrhosis on the expression of enzymes and transporters, abundance levels were compared across the three levels of disease severity (mild, moderate, and severe). A summary of the measured abundances is presented in Table S8, and the median values of the abundances of target protein for each group of samples are plotted in Figure S4. Figures 1–4 show the individual abundance values of CYP, UGT, non-CYP non-UGT, and transporter targets, respectively, with medians and 95% CI in each cirrhosis group, compared to the control. Figure S2 presents the fold change in the median abundance values for each enzyme or transporter at all stages of cirrhosis relative to the control.

The Kruskal–Wallis ANOVA test showed significant differences (*p* < 0.05) for most of the targets of interest, except UGT1A1 (*p* = 0.051), UGT1A3 (*p* = 0.34), MDR3 (*p* = 0.051), MRP4 (*p* = 0.25), BCRP (*p* = 0.78), ASBT (*p* = 0.18), and OATP1A2 (*p* = 0.11). For mild cirrhosis, median abundance was significantly lower than the control for only four proteins: CES1 (by 47%, *p* = 0.003*), FMO3 (by 37%, *p* < 0.001**), EPHX1 (by 41%, *p* = 0.005*), MGST3 (by 51%, *p* = 0.003*), MRP2 (54%, *p* < 0.001**), and OATP1B1 (by 54%, *p* < 0.001**). For the moderate cirrhosis group, some targets showed a statistically significant reduction from the control, by 40 to 50%, such as MGST1, MDR1, MRP3, OCT3, and ATP1A1. Several targets showed a more significant decline by up to 77% from the control group, including CYP3A4, 1A2, 2C8, 2C9, 2E1, 2D6, 2A6, 2J2, 4F2, UGT1A6/9, 2B4/7, CES1/2, FMO3, EPHX1, MGST3, OAT2/4, OCT1, MRP2/6, BSEP, OATP1B1, OATP2B1, NTCP, and MCT1. Only CYP2C19 showed a very high reduction of 98%. The largest reduction was observed with most of the targets in the severe grade of cirrhosis. The level of reduction ranged from 40 to 55% with MDR1, MRP3, ATP1A1, and OCT3, while a decline of 60 to 78% was noted for CYP2C8/9/18, 2D6, 2A6, UGT1A4/6, 2B4, 2B15, 1A9, CES2, FMO5, POR, MGST1/3, BSEP, MRP6, OAT2/4, OATP1B1, 2B1, NTCP, and MCT1. Furthermore, CYP3A4, 1A2, 2E1, 2J2, 4F2, 2C9/19, UGT2B7, CES1, FMO3, EPHX1, MRP2, and OCT1 showed 80 to 98% reduction in the disease group relative to that in the control. By contrast, CYP2B6 and OATP1B3 did not show a statistically significant change in any of the three cirrhosis groups compared to the control in spite of showing significant differences across groups with the Kruskal–Wallis ANOVA test (*p* < 0.05). For both targets, several of the measurements across the groups fell below the limit of quantification which precluded the detection of differences.

### Relative Distribution of Enzymes and Transporters in Cirrhotic Livers

The relative distribution of the enzymes and transporters was determined for each severity stage based on absolute abundance values. Although there were non-uniform changes, the rank-order of hepatic enzymes (CYPs and UGTs) was generally consistent in cirrhosis compared to the control with few exceptions (Figure S3A–C). CYP2C9 and CYP3A4 were the most abundant CYPs across all groups. Going from the control group to severe cirrhosis, CYP2E1 dropped down from the third most abundant CYP to the fifth rank. On the contrary, for UGTS, UGT1A1 ranked fifth in the control group and second in the mild and severe cirrhosis groups. UGT2B7 and 2B4 were the dominant UGTs in all groups. Non-CYP and non-UGT enzymes did not show major differences in their relative distribution between the control and diseased livers.

For transporters, the relative distributions were consistent for SLC transporters (Figure S3D), while for ABC transporters, the rank of P-gp was higher and MRP2 was lower in severe cirrhosis relative to that in the control (Figure S3E).

### Correlations of Transporter and UGT Abundance with Total Serum Bilirubin Levels

Enzymes and transporters are involved in the metabolism and elimination of endogenous substances, such as bilirubin; this is used to determine the severity of liver impairment. Therefore, the total serum bilirubin data obtained for each patient were correlated with corresponding UGT and transporter expression levels. The mean total serum bilirubin level (±SD) was 11 ± 6.1 for the control group, 18.2 ± 6.0 in mild, 36.8 ± 21.7 in moderate, and 132.7 ± 135.3 μmol/L in severe cirrhosis. The Spearman correlation analysis was used to investigate the relation between UGT or transporter abundances and log-transformed total bilirubin level in the serum (see the Supporting Information). UGT1A4/6/9 and UGT2B4/7/15 showed moderate negative correlations with log-transformed total bilirubin with *R*^2^ ranging from 0.3 to 0.4, *R*_s_ from −0.6 to −0.8, and *p* < 0.0006 (Figure S5).

Negative correlations between the transporter abundance and log-transformed total bilirubin were observed (*R*^2^ from 0.3 to 0.5; *R*_s_ from −0.5 to −0.8, *p* < 0.002) in the case of bile efflux transporters, MDR1, BSEP, MRP2, MRP3, and MRP6, and also uptake transporters, NTCP, MCT1, OCT1, OCT3, OATP2B1, OAT2, OAT4, and OATP1B1/1B3 (Figure S6).

### Effect of Etiology of Liver Cirrhosis on the Abundance of Enzymes and Transporters

**Figure 5:**
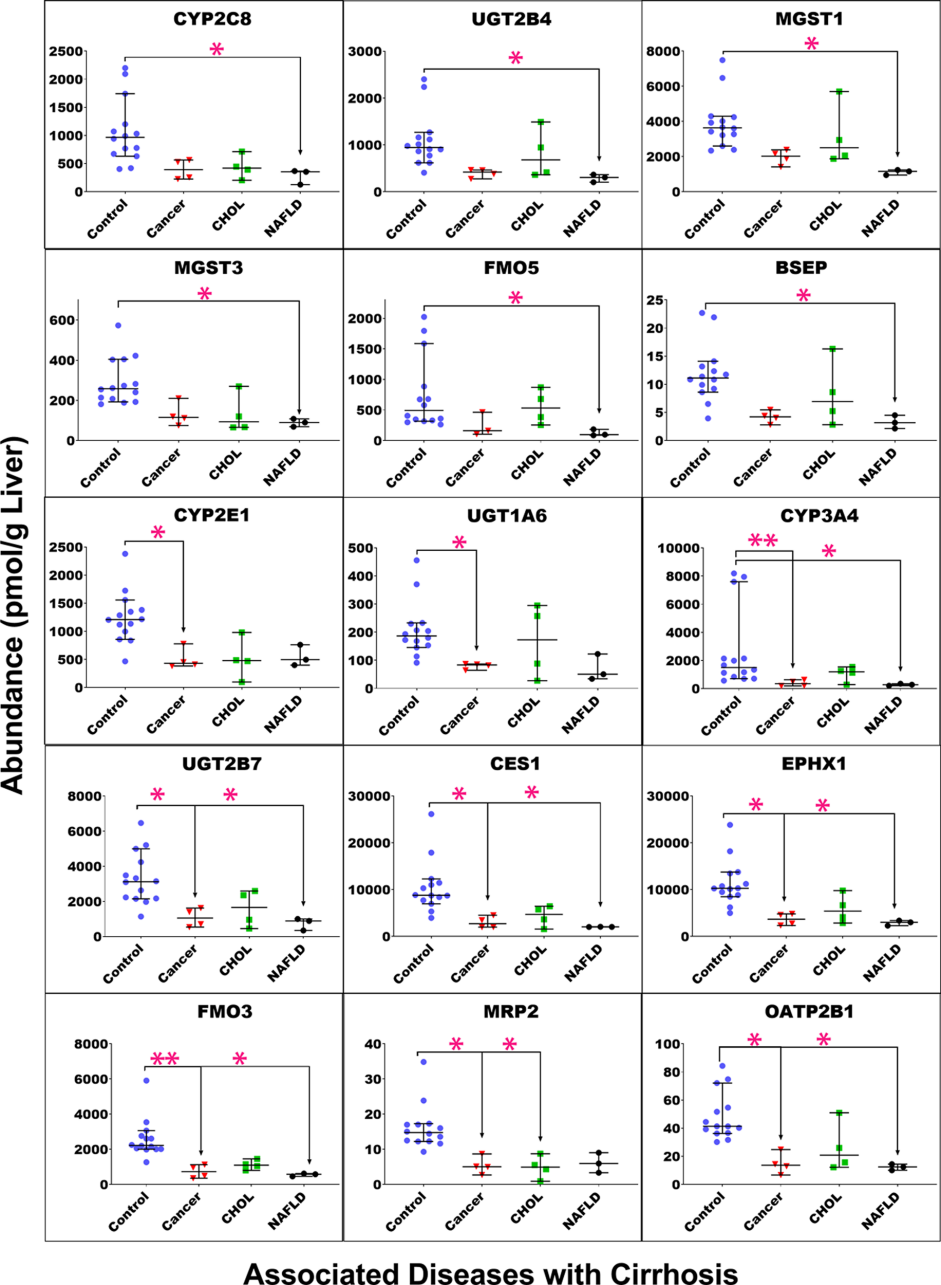
Individual abundance values of drug-metabolizing enzymes and transporters in moderate cirrhosis groups classified by the associated liver disease into cancer; CHOL, cholestasis; and NAFLD, non-alcoholic fatty liver disease, compared to the control group. Horizontal lines represent medians, and error bars are the 95% CIs. Stars represent comparisons that are statistically significant (*p < 0.0085 and **p < 0.0017).

To assess the effect of different etiologies, at the same degree of liver cirrhosis, on the expression of enzymes and transporters, data were compared for samples in the CP-B group. Cancer and NAFLD were associated with a more significant reduction in the levels of most targets, relative to the control, than cholestasis-related cirrhosis (Figure 5). Targets affected only by NAFLD-associated cirrhosis were CYP2C8, MGST1, MGST3, UGT2B4, FMO5, and BSEP, while targets that showed a significant reduction only with cancer-associated cirrhosis were CYP2E1 and UGT1A6. Targets significantly affected by both diseases (cancer and NAFLD) were CYP3A4, FMO3, UGT2B7, OATP2B1, EPHX1, and CES1. The only target showing a significant reduction in cholestasis-associated cirrhosis was MRP2; expression of this transporter was also reduced with cancer but not with NAFLD. Furthermore, the degree of difference across the disease groups was not statistically significant in the current study (Table S7).

### Impact of Applying the Generated Proteomic Data on the Performance of PBPK Models of Cirrhosis

**Figure 6:**
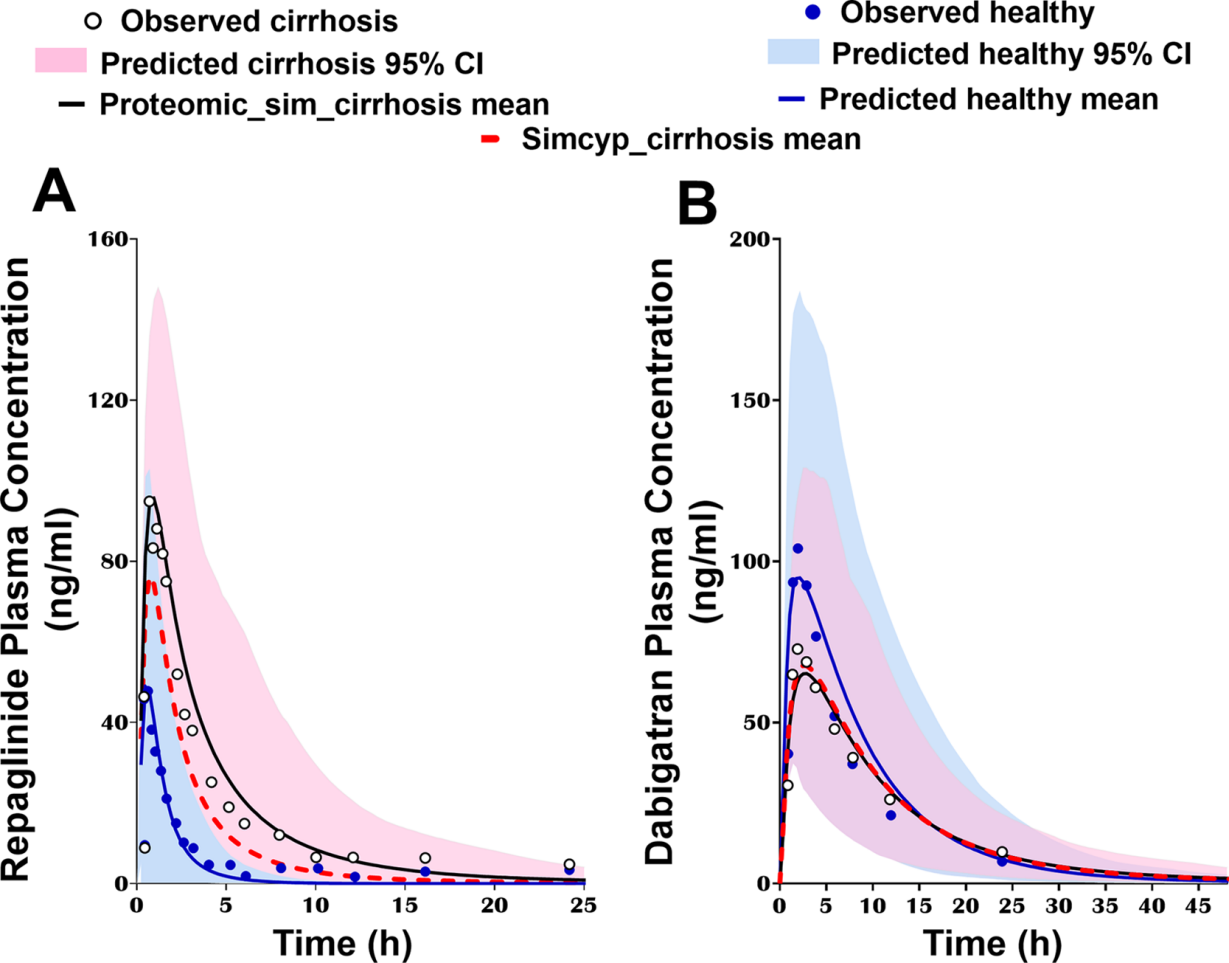
(A) Repaglinide- and (B) dabigatran-simulated plasma concentration–time profiles with changes in the abundance of metabolizing enzymes and transporters using proteomic data from the current study (Proteomic_sim_cirrhosis mean; solid black lines) and default settings in Simcyp V19 (Simcyp_cirrhosis mean; dotted red lines) in cirrhosis populations, compared to profiles in a healthy population (blue line). The corresponding observed data are presented for diseased (white circles) and healthy individuals (blue circles). 95% CI, 95% CI around the mean.

To simulate the impact of disease progression on drug PK, the proteomic data at different grades of severity were applied in PBPK models for repaglinide (in mixed CP-B and -C populations) and dabigatran etexilate (in CP-B population). For dabigatran simulations, both models (with proteomic changes in CES1/2 in cirrhosis relative to normal and the default Simcyp settings) were able to capture drug exposure in cirrhosis (<1% difference in AUC_pred_) (Figure 6). For repaglinide, the AUCR_pred_ using proteomic data from the current study was 4.9 compared to 2.8 with default Simcyp population settings (Table S9). The ratio of the predicted AUCR (AUCR_pred_) to the observed value (AUCR_obs_) was 1.19 using abundance data from the current study and 0.68 using default Simcyp population data.

**Figure 7:**
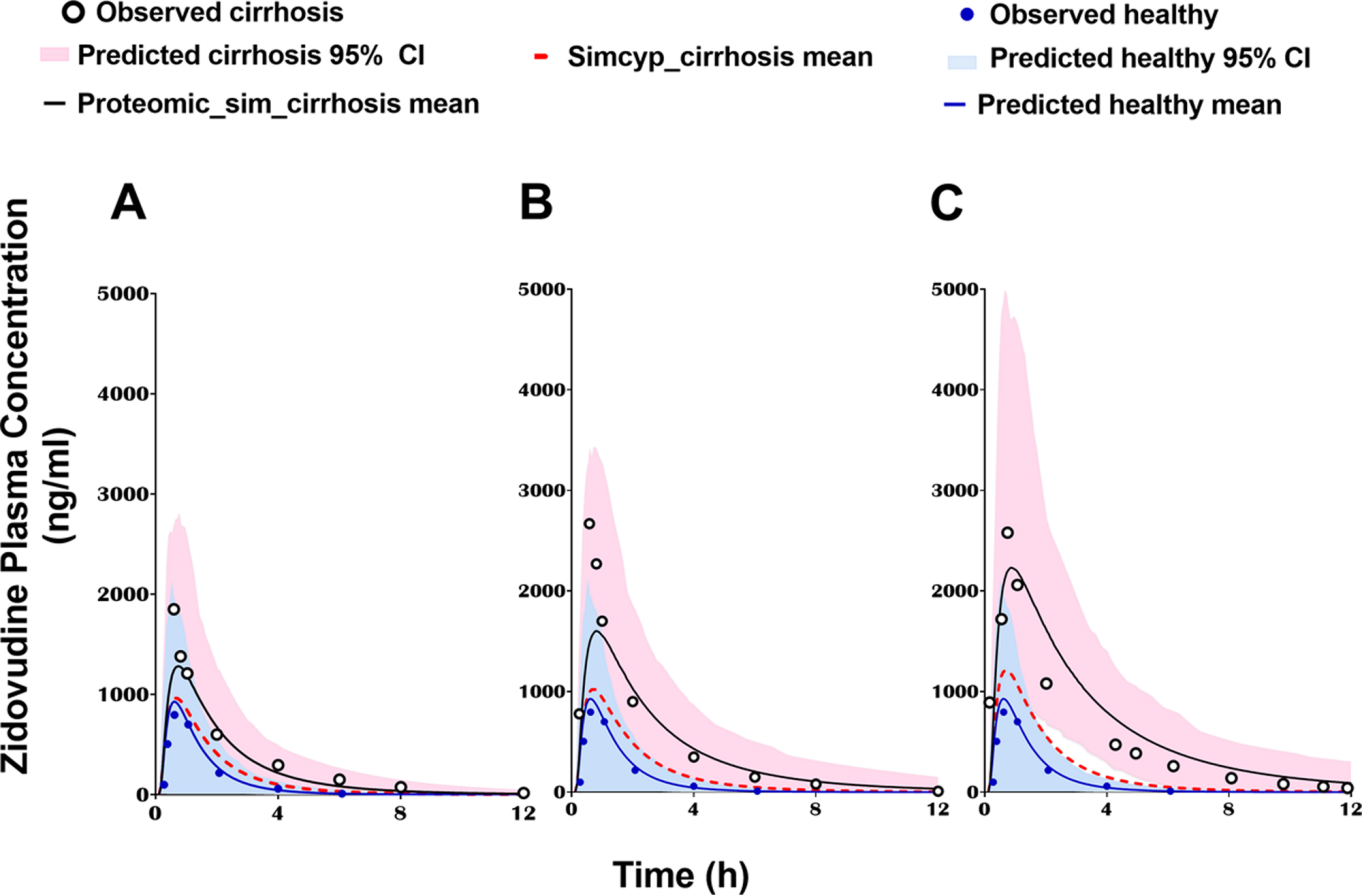
Zidovudine-simulated plasma concentration–time profile with changes in the abundance of metabolizing enzymes and transporters using proteomic data from the current study (Proteomic_sim_cirrhosis mean; solid black lines) and default settings in Simcyp V19 (Simcyp_cirrhosis mean; dotted red lines) in (A) mild, (B) moderate, and (C) severe cirrhosis populations, compared to the profile in a healthy population (blue line). The corresponding observed data are presented for diseased (white circles) and healthy individuals (blue circles). CI around the mean.

For zidovudine, the predicted simulations adjusted with proteomic data showed AUC levels within 2-fold of the observed data (Figure 7). Predicted-to-observed AUCR for CP-A, B, and C with proteomic data were 0.62, 0.97, and 1.2, respectively. By contrast, with default Simcyp abundance data, these values were outside the 2-fold range (0.38, 0.46, and 0.49, respectively), as shown in Table S9. Because non-parametric statistics was applied, we note that the intrinsic clearance values are scaled in disease populations using relative changes in median protein abundances generated in this study.

## Discussion

Heterogeneity in chronic liver disease and the degree of change in hepatic metabolic function are a challenge in the selection of effective drug dosing to patients with an impaired liver function. Specific alteration in the metabolic pathway by which a drug is eliminated is one of the contributing factors to this issue. This is because not all enzymatic reactions are affected by the liver disease equally as demonstrated by the current study.

In this study, quantitative LC–MS proteomics was used for the comprehensive characterization of changes in the expression of enzymes and transporters in three grades (mild, moderate, and severe) of liver cirrhosis. We observed a progressive decline in the abundance of enzymes and transporters with increasing severity of cirrhosis compared to the control livers, in line with the progressive decline in MPPGL, which we reported previously.^14^ The suppressed expression of enzymes and transporters, as reported in this study, is likely due to the downregulation of gene expression by inflammatory cytokines. This has been reported for several chronic inflammatory diseases, such as cirrhosis, rheumatoid arthritis, and cancer.^33−35^ Cytokines, such as IL-6 and TNFα, were reported to increase with cirrhosis progression which supports the link to downregulation of expression.^36^

The main advantage of using microsomal fractions (instead of homogenate or S9) is that enrichment of metabolism- and disposition-related proteins allows detection of the highest number of relevant proteins, even those expressed at low concentrations. Structural changes in diseased samples, such as scarring, fibrosis, and increased collagen in the extracellular matrix,^37^ may affect homogenization and membrane extraction. Several microsomal targets, such as CYP3A4, 1A2, 2A6, POR, UGT1A4, and UGT2B7, showed a comparable change in severe cirrhosis from the control to those reported by others.^11^ On the other hand, CYP2D6, 2E1, 2C8, 2C9, CES1, and CES2 were more affected by severe stages of cirrhosis in our study than reported in previous studies.^11^ The change in CYP2D6 was consistent with earlier reports.^5,38^ These differences might be attributed not only to differences in disease causes but also to the fact that previous reports did not classify samples based on full criteria of the CP scoring system (as we have done herein) and only considered transplantation to occur in severe stages of the disease, which is not always accurate. Therefore, this misclassification could increase the likelihood of including moderate, and possibly even mild, cases in the sample donors.^39^ The current study has reported, additionally, the change in earlier stages of cirrhosis (mild and moderate) as well as the severe stage.

Several older reports have claimed that phase II reactions are less affected by HI than oxidative phase I reactions.^40−42^ We now show that the expression of several UGTs, such as UGT1A6, 1A9, 2B4, and 2B7, is significantly (to the same degree as CYP enzymes) impaired by cirrhosis, especially in moderate to severe stages. We also quantified nine non-CYP and non-UGT metabolizing enzymes, resident in the endoplasmic reticulum, in all stages of cirrhosis severity, of which, MGST1, MGST3, and FMO5 are reported for the first time in cirrhosis. The NuncCAT is also capable of quantifying several sulfotransferases and ADH1, ALDH1A1, AOX, NAT, and EPHX2. These were not explicitly quantified here because they are cytosolic enzymes. The enrichment achieved by separating the microsomal fraction and previously reported for the same samples^14^ has allowed us to quantify CYP2B6 and UGT1A1 which have not been reported previously in cirrhotic livers using other non-enriched fractions such as the S9 fraction.^11^ Several measurements of CYP2B6 still fell below the limit of quantification, precluding detection of differences across groups.

We assessed changes in the relative distribution of enzymes in cirrhotic livers compared to the control as these changes might affect the impact of drug–drug interactions (DDIs) in cirrhosis compared to those in healthy populations. CYP2E1 was noticeably different as it showed a lower relative abundance in severe cirrhosis compared to other groups. This non-uniformity in disease impact was similarly observed in UGT expression and consequently the relative distribution of UGTs in health and disease. As expected,^11,17,43,44^ UGT2B7 and UGT2B4 were the most abundant enzymes of this class in the control samples. The relative abundance of UGT1A1 was, however, higher in mild and severe stages of cirrhosis compared with that in the controls as it is barely affected by the disease. These changes might be important for drugs cleared by multiple pathways, with expected changes in the relative contribution of each pathway (*f*_m_) with disease progression and the response to metabolic DDIs.^45,46^

This study and other recent reports suggest variability in the impact of cirrhosis on the expression of transporters according to the disease severity and the underlying pathophysiology (viral, alcoholic, and biliary diseases).^12,47,48^ Drozdzik *et al.*(47) showed a progressive decline in the expression of NTCP, OATP1B1/2B1, OCT1, and MRP2 in line with our findings, but we also showed a progressive decline in the expression of BSEP, MRP3, OAT2, OCT3, and OATP1B3 in cirrhosis, which they did not. It is worth noting that associated diseases to cirrhosis in the two sets of samples are different (ours includes NAFLD, cancer, and cholestasis, while Drozdzik *et al.* used samples with viral, cholestatic, auto-immune hepatitis, and alcohol-associated cirrhosis), which might have an impact on the degree of change. The effect of underlying conditions in the patient cohort was further investigated at the same level of severity (moderate cirrhosis). Generally, NAFLD and cancer-related cirrhosis had a higher impact on the expression of enzymes and transporters compared with cholestasis. This difference in impact was previously reported to affect MPPGL in the same samples.^14^ Molecular differences in the pathophysiology between hepatocellular and cholestatic cirrhosis are thought to be key players in the relative downregulation pattern for metabolism-related proteins, response to inflammatory mediators, and mRNA expression among different cirrhosis causes.^49,50^ The current CP classification system does not distinguish different causes or pathophysiologies of cirrhosis.^51^ Additional studies with larger numbers of samples per disease cause are recommended to better elucidate differences among groups.

Bilirubin level is used as a liver function test and as a component of the CP scoring system.^52^ Bilirubin is released into the blood after the breakdown of hemoglobin, conjugated in the liver by UGTs, and then excreted into the bile by transporters.^53^ Elevated levels (above the normal limit of 20.5 μmol/L) occur in different conditions, including liver disease.^54^ Progressive elevation with disease severity was observed in our data. The negative correlations between total bilirubin and some UGT levels can be attributed to the decreased ability of the diseased livers to conjugate bilirubin, leading to hepatic jaundice. Correlations between transporter abundances and total serum bilirubin can also be explained mechanistically; both efflux and uptake transporters have roles in bilirubin disposition. The drop in their expression leads to hyperbilirubinemia, which is common in patients with cirrhosis and can be used as a predictor of poor prognosis.^55^ These uptake and canalicular efflux transporters play key roles in biliary secretion and enterohepatic recycling of some drugs, which, when taken into account in PBPK models, can help in predicting their plasma levels, and ultimately, dose adjustment for these drugs in health and disease.^56−58^ It is also important to note that the level of bilirubin is determined not just by its elimination but also by its synthesis, and therefore, a good correlation with transporter abundance might indicate that there was little change in synthesis or the change was comparable in all patients.

PBPK modeling aims to optimize drug therapy regimens for patients in clinical practice^59^ by predicting drug kinetics and selecting appropriate drug dosage regimens. The use of PBPK is particularly useful in studying changes in PKs associated with special physiological populations, such as HI;^60^ however, system parameters specific to these populations have generally been lacking. The reduction in the expression of metabolizing enzymes is typically associated with reduced drug clearance and increased AUC.^6^ However, the degree of this increase is different from one drug to another according to the sensitivity of the drug’s kinetics to changes in expression and the impact of other physiological changes in cirrhosis, such as plasma protein levels, binding, and hepatic blood flow.^61^ These factors can sometimes affect drug exposure in the opposite direction to changes in enzyme expression. Therefore, direct correlations cannot be performed and PBPK modeling and simulation are required to account for these factors simultaneously. Modeling and simulation platforms have hitherto employed protein expression data generated by Western blotting,^5^ but these data have recently started to be supplanted by data generated by state-of-the-art proteomics.^62^ We used our data to simulate the impact of changes in the abundance of liver enzymes on exposure of a cirrhotic population to repaglinide, dabigatran, and zidovudine. The choice of the drugs aimed to cover one or more proteins from each group (CYPs, UGTs, non-CYP non-UGT enzymes, and transporters) while prioritizing drugs that have clinical data in both healthy and cirrhosis patients and have a verified model that is either available in the simulator’s library or was reported in a previous publication. The performance of the models adapted with the current proteomic data was compared with the output from Simcyp default cirrhosis settings which were based on a combination of available Western blotting abundance and *in vitro* and *in vivo* activity studies.^5^ Simcyp default settings in cirrhosis populations did not account for changes in OATP transporters and non-CYP enzyme abundances. Therefore, it was clear that when proteomic data were applied for repaglinide and zidovudine, the performance of the models was improved. However, for dabigatran, a similar outcome to the default Simcyp output profile was observed. This can be attributed to the low sensitivity of active drug exposure to changes in the expression of carboxyesterases.

## Conclusions

This study demonstrated, for the first time, a gradual decline in the expression of enzymes and transporters with the progression of cirrhosis severity. The rate of this decline was specific to each target protein. The impact of the underlying condition was most significant in the cases of cancer and NAFLD. However, one limitation of this study is the small sample size in each disease severity and etiology group. Introducing specific proteomic data related to changes due to cirrhosis into the population parameters of the PBPK models can improve the predictive performance of these models in HI populations. The study provides some biological reasons behind the lack of a single drug dose-adjustment formula in cirrhosis and demonstrates the utility of proteomics-informed PBPK modeling for drug-specific dose adjustment in liver cirrhosis.

## Supporting Information Available

Demographics of sample donors, details on digestion and analysis protocols and statistics; surrogate peptides for each target, physiological parameters implemented in the simulator, demographics of subjects in the simulated trials, scalars applied to each sample, absolute abundances values of all targets, impact of disease etiology on the abundances of most targets; levels of experimental variabilities, fold changes in the protein abundance with disease severity, pie charts showing the relative abundance distribution of targets; and correlations between total bilirubin and UGTs or transporters expressions (PDF)

## Abbreviations

DMET: drug-metabolizing enzymes and drug transporters
LC–MS: liquid chromatography–mass spectrometry
QconCAT: quantification concatemer
PBPK: physiologically based pharmacokinetics
HI: hepatic impairment
CP: Child–Pugh
HLM: human liver microsomes
NAFLD: non-alcoholic fatty liver disease
CYP: cytochrome P450
UGT: uridine-5′-diphospho-glucuronosyltransferase
CL_int_: intrinsic clearance
AUC: area under the concentration–time curve
AUCR: the ratio of the area under the concentration–time curve in diseased to healthy populations
