# Supplementary material for: Non-uniformity of Changes in Drug-Metabolizing Enzymes and Transporters in Liver Cirrhosis: Implications for Drug Dosage Adjustment
Source: Mol Pharm. 2021 Aug 24;18(9):3563–77. doi: 10.1021/acs.molpharmaceut.1c00462 (PMC8424631; doi:10.1021/acs.molpharmaceut.1c00462)
Supplement: Supplementary file 1 — mp1c00462_si_001.pdf [file mp1c00462_si_001.pdf]

Molecular Pharmaceutics

Supplementary Information

Non-uniformity of Changes in Drug-Metabolizing Enzymes and Transporters in Liver Cirrhosis: Implications for Drug Dosage Adjustment

Eman El-Khateeb, Brahim Achour, Zubida M. Al-Majdoub, Jill Barber, Amin Rostami-Hodjegan

Table S1. Demographic and clinical information for the individual donors of control samples.

Serial	Sample ID	Date of surgery	Age	sex	PT (sec)	Albumin level (g/L)	Weight (Kg) *	Height (m) *	Total bilirubin	General diagnosis
**1**	2759	12/12/16	81	M	11.3	34	82	1.72	11	CRC
**2**	2721	06/12/16	36	M	11.5	39	61.5	1.696	18	CRC
**3**	2841	28/12/16	57	M	12.3	40	84	1.74	9	CRC
**4**	0103	16/01/17	81	M	21.6	38	75	1.67	16	CRC
**5**	2847	30/12/16	48	F	12.2	43	67.8	1.619	10	SCC
**6**	0044	09/01/17	83	F	10.6	39	62.3	1.637	6	CRC
**7**	761	20/04/17	73	M	12.7	35	94.9	1.638	6	HCC
**8**	713	13/04/17	57	F	12.1	42	65.9	1.73	9	CRC
**9**	502	14/03/17	77	M	11.9	38	112.5	1.71	9	CRC
**10**	0125	19/01/17	62	M	10.9	38	69.7	1.7	7	CRC
**11**	0336	16/02/17	71	F	10.5	34	76	1.53	8	GIST
**12**	484	13/03/17	80	M	21.9	24	71	1.81	28	CRC
**13**	0322	14/02/17	71	M	10.8	43	93.6	1.715	11	CRC
**14**	2809	20/12/16	52	M	10.1	42	88	1.735	6	CRC

PT, prothrombin time; *measured at time of surgery; HCC, hepatocellular carcinoma; CRC, colorectal cancer; SCC, squamous cell carcinoma; GIST, gastrointestinal stromal tumor.

Table S2. Demographic and clinical information for the individual donors of cirrhosis liver samples with associated Child-Pugh classification.

Serial for each group	Sample ID	Date of surgery DD/MM/YY	Age	sex	PT (sec)	Albumin level (g/L)	Total bilirubin (µmol/L)	Weight (Kg) [a]	Height (m) [a]	Ascites volume[b]	HE grade [c]	General diagnosis	CP class (Score)
**1**	0974	19/05/17	56	M	18.1	37	8	117.5	1.75	Severe	1-2	SH	B(9)
**2**	1982	17/08/16	63	F	18	25	30	70.7	1.57	Mild	1-2	NAFLD	B(9)
**3**	1570	10/06/16	62	F	16.6	32	26	72.1	1.52	Moderate	1-2	NAFLD	B(9)
**4**	0549	22/03/17	59	M	18.8	28	53	89.2	1.72	Mild	1-2	NAFLD	C(10)
**5**	0355	17/02/17	67	F	17.9	26	81	82.2	1.64	Moderate	None	NAFLD	C(11)
**6**	0863	08/05/17	51	F	19.8	23	78	89.2	1.6	Mild	None	NAFLD	C(11)
**7**	2728	07/12/16	66	F	24.9	32	29	82.6	1.6	Moderate	1-2	NAFLD	C(10)
**8**	1571	11/06/16	46	F	14.7	25	51	78.6	1.58	Mild	1-2	NAFLD	C(11)
**1**	2403	21/10/16	57	M	14.4	29	16	89	1.803	None	None	HCC & HCV	A(6)
**2**	0955	18/05/17	63	M	15	36	18	89.2	1.73	None	None	HCC & alcoholic SH	A(5)
**3**	1963	13/08/16	68	M	13.6	27	28	113.45	1.77	None	1-2	HCC & alcoholic	B(8)
**4**	1745	14/07/16	67	M	15.2	27	46	92	1.7	Mild	None	HCC & NAFLD	B(9)
**5**	2431	27/10/16	69	M	15.7	28	30	95	1.79	None	None	HCC & HCV	A(6)
**6**	2408	22/10/16	51	M	18.9	31	44	88.5	1.85	Mild	None	HCC & HCV	B(8)
**7**	1926	10/08/16	63	M	17	33	97	82.3	1.74	None	1-2	HCC & NAFLD	B(9)
**8**	3688	12/11/15	65	F	13.8	40	14	84	1.63	None	None	HCC & alcoholic SH	A(5)
**9**	1228	06/12/17	55	F	14.3	29	13	78.6	1.62	None	None	HCC	A(6)
**1**	997	21/04/16	59	M	19.7	13	63	100.8	1.82	Severe	1-2	CHOL	C(14)
**2**	0746	19/04/17	67	M	13.7	24	58	76.4	1.6	None	1-2	PBC	C(10)
**3**	0147	25/01/17	57	M	15.3	19	243	72.3	1.74	Mild	1-2	CHOL	C(11)
**4**	2682	02/12/16	56	F	15.2	40	75	67.3	1.62	Severe	1-2	CHOL	C(11)
**5**	2500	07/11/16	63	F	11.9	30	82	80.15	1.64	Mild	1-2	PBC	C(10)
**6**	2306	11/10/16	60	M	16.9	22	326	83.2	1.76	Mild	1-2	PSC	C(11)
**7**	2159	15/09/16	39	M	14.2	30	49	80.8	1.90	Mild	None	PSC	B(8)
**8**	2136	12/09/16	54	M	19.1	30	484	79.7	1.75	Moderate	1-2	PSC	C(12)

**9**	1684	11/07/16	69	F	15.4	28	102	78.6	1.67	Mild	1-2	CHOL & PBC	C(10)
**10**	1429	18/07/17	57	M	15.2	37	37	69	1.75	Mild	None	PSC	B(7)
**11**	0544	21/03/17	70	M	13.3	30	25	87.8	-	None	1-2	PSC	B(7)
**12**	2020	24/08/16	59	F	15.9	29	26	43.95	1.5	Severe	None	PBC	B(9)
**13**	0819	05/02/17	75	M	13.2	41	18	79.5	1.6	None	None	CHOL	A(5)
**1**	1509	04/06/16	57	M	14.4	33	18	74.95	1.75	Moderate	1-2	Alcoholic SH	B(9)
**2**	1545	09/06/16	69	F	20.9	50	44	62.7	1.55	Moderate	None	Alcoholic SH	B(9)

PT, prothrombin time; CP, Child-Pugh; HCC, hepatocellular carcinoma; NAFLD, non-alcoholic fatty liver disease; CHOL, cholestasis; PBC, primary biliary cirrhosis; PSC, primary sclerosing cholangitis, SH, steatohepatitis; [a] measured at time of surgery; [b] controlled with diuretics recorded as mild ascites, [c] controlled with rifaximin / lactulose recorded as grade 1-2.

Table S3. Targets and their surrogate peptides in each QconCAT standard, NuncCAT, MetCAT and TransCAT

NuncCAT	
**Target**	**Peptide Sequence**
**CES1**	EGYLQIGANTQAAQK§
	FLSLDLQGDPR
**CES2**	ADHGDELPFVFR
	SFFGGNYIK§
	TTHTGQVLGSLVHVK
**FMO3**	LVGPGQWPGAR§
	NNLPTAISDWLYVK
**FMO5**	WATQVFK§
	TDDIGGLWR
	LTHFIWK
**EPHX1**	IIPLLTDPK§
	FSTWTNTEFR
**POR**	QYELVVHTDIDAAK
	YYSIASSSK
	IQTLTSSVR§
**MGST1**	VFANPEDCVAFGK§
	IYHTIAYLTPLPQPNR
**MGST2**	HLYFWGYSEAAK
**MGST 3**	IASGLGLAWIVGR§
	VLYAYGYYTGEPSK
**UGT2B17**	WTYSISK§
	GHEVIVLTSSASILVNASK
	SVINDPIYK
**ADH1A***	GAILGGFK
	NDVSNPQGTLQDGTSR
	KPIHHFLGISTFSQYTVVDENAVAK
**ADH1B ***	AAVLWEVK
	GAVYGGFK
**ADH1C**	FSLDALITNILPFEK

MetCAT	
**Target**	**Peptide Sequence**
**CYP1A2**	ASGNLIPQEK§
	YLPNPALQR§
**CYP2A6**	DPSFFSNPQDFNPQHFLNEK
	GTGGANIDPTFFLSR§
**CYP2B6**	ETLDPSAPR
	GYGVIFANGNR§
**CYP2C18**	GSFPVAEK
	SLTNFSK§
**CYP2C19**	GHFPLAER
**CYP2C8**	SFTNFSK§
**CYP2C9**	GIFPLAER§
	LPPGPTPLPVIGNILQIGIK
**CYP2D6**	AFLTQLDELLTEHR
	DIEVQGFR§
**CYP2E1**	FITLVPSNLPHEATR
	GIIFNNGPTWK§
**CYP2J2**	FEYQDSWFQQLLK
	VIGQGQQPSTAAR§
**CYP3A4**	EVTNFLR
	LSLGGLLQPEK§
**CYP3A43**	YIPFGAGPR
**CYP3A5**	DTINFLSK§
	YWTEPEEFRPER
**CYP3A7**	FGGLLLTEK§
	FGGLLLTEKPIVLK
	FNPLDPFVLSIK
**CYP4F2**	HVTQDIVLPDGR§
**UGT1A1**	DGAFYTLK§
	TYPVPFQR
**UGT1A3**	HVLGHTQLYFETEHFLK

TransCAT	
**Target**	**Peptide Sequence**
**P-gp, MDR1**	FYDPLAGK§
	AGAVAEEVLAAIR
**BSEP**	STALQLIQR
	AADTIIGFEHGTAVER§
**MDR3**	IATEAIENIR
	GAAYVIFDIIDNNPK§
**MRP2**	LTIIPQDPILFSGSLR§
	YLGGDDLDTSAIR
	AFEHQQR
**MRP3**	AEGEISDPFR
	IDGLNVADIGLHDLR§
**MRP4**	AEAAALTETAK
	APVLFFDR§
**MRP6**	SSLPSALLGELSK
	APETEPFLR§
	SSLASGLLR
**BCRP**	VIQELGLDK
	SSLLDVLAAR§
	ENLQFSAALR
**ATP1A1**	IVEIPFNSTNK
	SPDFTNENPLETR§
**Cadherin-17**	AENPEPLVFGVK
	QNSRPGK
**Cadherin-23**	ATDADEGEFGR
	DAYVGALR
**OST-α**	YTADLLEVLK
	VGYETFSSPDLDLNLK
**OST-β**	DHNSLNNLR
	ETPEVLHLDEAK

**ALDH1A1 ***	IFVEESIYDEFVR
	IFINNEWHDSVSGK
	TIPIDGNFFTYTR
**AOX1 ***	LILNEVSLLGSAPGGK
	GLHGPLTLNSPLTPEK
	VFFGEGDGIIR
**NAT1 ***	DNTDLIEFK
	NYIVDAGFGR
**NAT2***	TLTEEEVEEVLK
	DNTDLVEFK
**SULT1E1***	KPSEELVDR
	NHFTVALNEK
**SULT1A1**	VHPEPGTWDSFLEK
**SULT1A2**	VYPHPGTWESFLEK
**SULT2A1***	DEDVIILTYPK
	TLEPEELNLILK
**TPMT***	NQVLTLEEWQDK
	TSLDIEEYSDTEVQK
**EPHX2 ***	GLLNDAFQK
	WLDSDAR

**UGT1A4**	YLSIPTVFFLR[§]
	GTQCPNPSSYIPK[§]
	YIPCDLDFK
**UGT1A6**	SFLTAPQTEYR
	VSVWLLR[§]
**UGT1A9**	AFAHAQWK[§]
	ESSFDAVFLDPFDNCGLIVAK
**UGT2B4**	ANVIASALAK
	FSPGYAIEK[§]
**UGT2B7**	ADVWLIR[§]
	TILDELIQR
**UGT2B10**	GHEVTVLASSASILFDPNDSSTLK
**UGT2B11**	GHEVTVLASSASILFDPNDASTLK
**UGT2B15**	SVINDPVYK
	ASGNLIPQEK
	WIYGVSK[§]

**OCT-1**	MLSLEEDVTEK
	GVALPETMK[§]
	ENTIYLK
**OCT-2**	GIALPETVDDVEK
	FLQGVFGK
**OCTN2**	TWNIR
	DYDEVTAFLGEWGPFQR
**OAT2**	WLLTQGHVK
	NVALLALPR[§]
**OAT4**	DTLTLEILK
	ISLLSFTR[§]
**MATE-1**	GGPEATLEVR
	DHVGYIFTTDR
**OATP1A2**	EGLETNADIIK
	IYDSTTFR[§]
**OATP1B1**	YVEQQYGQPSSK[§]
	MFLAALSLSFIAK
	LNTVGIAK
**OATP1B3**	NVTGFFQSLK[§]
	IYNSVFFGR
**OATP2B1**	VLLQTLR
	SSPAVEQQLLVSGPGK[§]
**OATP4C1**	HLPGTAEIQAGK
	SPEPSLPSAPPNVSEEK
	DFPAALK
**NTCP**	GIYDGDLK[§]
	GIVISLVLVLIPCTIGIVLK
	AHLWKPK
**PEPT-1**	GNEVQIK
	TLPVFPK
	HTLLVWAPNHYQVVK
**ASBT**	IAGLPWYR[§]
	LWIIGTIFPVAGYSLGFLLAR
**MCT-1**	SITVFFK[§]
	DLHDANTDLIGR
**OATP4A1**	YEVELDAGVR
	ILGGIPGPIAFGWVIDK

* Represent targets mainly located in the cytosolic fraction; [§]Peptides used for the quantification of the targets in the current study

Table S4. Physiological parameters for healthy individuals and cirrhosis patients within Simcyp Simulator V19.

Liver condition	Healthy Control	Mild Impairment	Moderate Impairment	Severe Impairment
**Simcyp-population**	HV	CP-A	CP-B	CP-C
**Gastric residence time (hr) (fasted/fed)/colon transit time**	0.4/1.0/12	0.48/1.2/24	0.55/1.38/24	0.6/1.5/24
**Albumin/a1-AG/Haematocrit (ratio to HV, male)**	1 / 1/1	0.8 /0.9/0.9	0.7 /0.8/0.8	0.6/0.6/0.8
**CYP3A4 abundance in the liver (pmol/mg)**	137	108	56	31.7
**CYP2C8 abundance in the liver (pmol/mg)**	24	16.6	12.5	7.92
**OATP1B1 abundance in the liver (pmol/mg)**	3.1	3.1	3.1	3.1
**CES1/CES2 abundance in the liver (pmol/mg)**	ND	ND	ND	ND
**CYP3A4 abundance in the intestine (pmol/mg)**	65.4	65.4	39.9	23.6
**Liver Volume (L)**	1.65	1.47	1.17	1.0
**Liver Q (Arterial/Portal), (% cardiac output)**	6.5/19	9.2/17.3	10.6/13.6	12.5/10.5

ND, No data are available; CP, Child-Pugh score; HV, healthy volunteer; Q, blood flow.

Table S5. Demographic data of the healthy and cirrhosis subjects used for repaglinide, dabigatran etexilate and zidovudine simulations.

Drug	Parameter	Cirrhosis patients	Healthy subjects	References
**Repaglinide**	Mean age in years (range)	52.9 (37- 62)	53.2 (42- 62)	[1]
	Proportion of females	0	0	
	Weight (kg) ± SD	86.8 ± 13.9	78.5 ± 12	
	Number of participants	12 (9 CP-B, 3 CP-C)	12	
**Dabigatran etexilate**	Mean age in years (range)	55.2 (41-68)	54.9 (38-66)	[2]
	Proportion of females	0.417	0.417	
	Weight (kg) ± SD	80.6 ± 17.7	82.3 ± 18.7	
	Number of participants	12 (all are CP-B)	12	
**Zidovudine**	Mean age in years (range)	CP-A 57 (54-59), CP-B 52 (43-60), CP-C 53 (46-63)	45 (26-62)	[3]
	Proportion of females	0	0	
	Weight (kg) ± SD	CP-A 71 ± 6, CP-B 73 ± 16, CP-C 63 ± 15	70 ± 10	
	Number of participants	3 (CP-A), 5 (CP-B), 6 (CP-C)	6	

SD, standard deviation; CP-B and CP-C, Child-Pugh grades B (moderate cirrhosis) and C (severe cirrhosis).

Table S6. MPPGL values for each sample used for scaling up abundances in pmol/mg microsomal protein to pmol/g liver tissue.

Serial	Sample ID	Type<br>[CP class (grade), disease cause]	MPPGL<br>(mg microsomes/g liver)
**1**	2759	HN	31.4
**2**	2721	HN	33
**3**	2841	HN	30.4
**4**	0103	HN	40.7
**5**	2847	HN	63.6
**6**	0044	HN	34.9
**7**	761	HN	61.8
**8**	713	HN	49.5
**9**	502	HN	34.9
**10**	0125	HN	37.9
**11**	0336	HN	35.4
**12**	484	HN	39.2
**13**	0322	HN	36.6
**14**	2809	HN	62.7
**15**	0974	B(9), NAFLD	28.5
**16**	1982	B(9), NAFLD	30.8
**17**	1570	B(9), NAFLD	36
**18**	0549	C(10), NAFLD	25.9
**19**	0355	C(11), NAFLD	25.1
**20**	0863	C(11), NAFLD	20.9
**21**	2728	C(10), NAFLD	20
**22**	1571	C(11), NAFLD	35
**23**	2403	A(6), Cancer	39.9
**24**	0955	A(5), Cancer	32.8
**25**	1963	B(8), Cancer	22.4
**26**	1745	B(9), Cancer	12.9
**27**	2431	A(6), Cancer	30
**28**	2408	B(8), Cancer	21
**29**	1926	B(9), Cancer	24.8
**30**	3688	A(5), Cancer	22
**31**	1228	A(6), Cancer	25.6
**32**	997	C(14), CHOL	28.1
**33**	0746	C(10), CHOL	18.5
**34**	0147	C(11), CHOL	29.5
**35**	2682	C(11), CHOL	38.3
**36**	2500	C(10), CHOL	37.2
**37**	2306	C(11), CHOL	40.9
**38**	2159	B(8), CHOL	42.4
**39**	2136	C(12), CHOL	41.7
**40**	1684	C(10), CHOL	34
**41**	1429	B(7), CHOL	49.1
**42**	0544	B(7), CHOL	44.8
**43**	2020	B(9), CHOL	35
**44**	0819	A(5), CHOL	42.2
**45**	1509	B(9), Alcoholic SH	30.2
**46**	1545	B(9), Alcoholic SH	32.4

CP, Child-Pugh; HN, histologically normal liver tissue, NAFLD, non-alcoholic fatty liver disease; CHOL, cholestasis; SH, steatohepatitis

Table S7. Comparison of the impact of disease associated with cirrhosis (cancer, cholestasis, and NAFLD) on the abundance of enzymes and transporters relative to normal control.

Groups / Target proteins	**ANOVA (Control and 3 diseased groups)**	**Control *vs* Cancer-cirrhosis % change in medians (*P*-value)**	**Control *vs* Cholestasis-cirrhosis % change in medians (*P*-value)**	**Control *vs* NAFLD-cirrhosis % change in medians (*P*-value)**	**ANOVA (3 diseased groups)**
CYP2A6	0.012^	-53 (0.018)	-52 (0.039)	-72 (0.017)	0.76
**CYP2C8**	0.003^	-60 (0.014)	-57 (0.026)	-63 (0.003*)	0.5
CYP2C9	0.013^	-59 (0.019)	+2 (0.9)	-63 (0.01)	0.051
CYP2C18	0.35	-70 (NA)	+26 (NA)	-53 (NA)	0.17
**CYP2E1**	0.002^	-64 (0.003*)	-60 (0.012)	-59 (0.024)	0.87
**CYP3A4**	0.003^	-77 (0.001**)	-20 (0.33)	-82 (0.003*)	0.11
CYP4F2	0.026^	-77 (0.017)	-54 (0.37)	-81 (0.02)	0.18
UGT1A1	0.05	-23 (NA)	-27 (NA)	-57 (NA)	0.49
UGT2B15	0.19	-49 (NA)	-39 (NA)	-54 (NA)	0.94
**UGT1A6**	0.013^	-55 (0.008*)	-7 (0.45)	-73 (0.016)	0.54
UGT1A9	0.023^	-52 (0.013)	-15 (0.92)	-63 (0.03)	0.085
**UGT2B4**	0.006^	-55 (0.015)	-28 (0.35)	-68 (0.003*)	0.14
**UGT2B7**	0.003^	-66 (0.008*)	-47 (0.059)	-72 (0.004*)	0.55
**CES1**	0.001^	-69 (0.002*)	-46 (0.02)	-77 (0.005*)	0.58
**FMO3**	0.001^	-67 (0.001**)	-51 (0.024)	-74 (0.002*)	0.09
**FMO5**	0.22^	-67 (0.08)	+8 (0.87)	-80 (0.006*)	0.06
**EPHX1**	0.001^	-64 (0.003*)	-47 (0.048)	-71 (0.003*)	0.29
**MGST1**	0.002^	-44 (0.013)	-31 (0.2)	-68 (0.001**)	0.023^ (NS)
**MGST3**	0.004^	-55 (0.024)	-64 (0.018)	-65 (0.006*)	0.62
POR	0.004^	-60 (0.009)	14 (0.58)	-62 (0.01)	0.046^ (NS)
**BSEP**	0.008^	-62 (0.01)	-38 (0.224)	-72 (0.007*)	0.29
MDR3	0.009^	-80 (0.12)	-23 (0.57)	-67 (0.098)	0.023^ (NS)
**MRP2**	0.001^	-66 (0.002*)	-67 (0.002*)	-60 (0.017)	0.74
MRP3	0.037^	-45 (0.015)	-44 (0.088)	-62 (0.1)	0.97
ATP1A1	0.032^	-42 (0.021)	-46 (0.039)	-43 (0.093)	0.82
OAT2	0.015^	-65 (0.036)	-74 (0.013)	-64 (0.051)	0.48

**OATP2B1**	0.002^	-67 (0.003*)	-50 (0.068)	-70 (0.004*)	0.32

NAFLD, non-alcoholic fatty liver disease; ^, statistically significant difference with Kruskal-Wallis ANOVA test ($p < 0.05$); (-) and (+) signs refer to decreased or increased abundance from control, respectively; *, statistically significant difference in pairwise comparison ($p < 0.008$); **, statistically significant pairwise comparison ($p < 0.0017$); NS, no statistical significance with post hoc test; NA, not applicable pairwise comparison as the ANOVA test showed no statistical significance ($p > 0.05$).

Table S8. Absolute abundance in pmol/g liver tissue of drug-metabolizing enzymes and transporters in normal controls and cirrhosis samples of various severities and causes.

Target	Normal control pmol/mg liver tissue			Mild (CP-A) cirrhosis pmol/mg liver tissue			Moderate (CP-B) cirrhosis pmol/mg liver tissue			Severe (CP-C) cirrhosis pmol/mg liver tissue		
	Mean ± SD	Median [CI]		Mean ± SD	Median [CI]		Mean ± SD	Median [CI]		Mean ± SD	Median [CI]	
CYP1A2	795.8±531	**665.8**	[359-1418]	**318.5±289**	**248.8**	[90-869]	272±180.4	**256.5**	[83-454]	99.7±72.4	**88.4**	[20.6-187]
CYP2A6	987.6±758	**693**	[368-1265]	**352±158**	**305.5**	[225-634]	298.8±140	**312**	[178-465]	221±129.6	**191**	[146-351]
CYP2B6	166±71	**166**	[88-304]	**98±4**	**98**	[96-101]	84±42	**82**	[25.5-140]	82.8±57	**56.7**	[45-110]
CYP2C18	28±22.8	**17.4**	[14.7-43.7]	**15±8**	**19**	[3.2-21.6]	10.4±8	**8**	[2.4-23.8]	7±4.4	**7**	[2.2-11]
CYP2C19	272±312	**126.6**	[34.7-601.8]	4.7±ND	**4.7**	ND	10.5±16	**1.3**	[0.1-39.8]	15.5±26	**5.8**	[1.8-68.5]
CYP2C8	1066.6±569	**965.6**	[630-1741]	**686±188**	**702**	[473-960]	362±165.5	**353**	[224-528]	252.8±163	**234.4**	[112-341]
CYP2C9	5124±2402	**4825.4**	[2885-8618]	**3450±925**	**3327**	[2139-4638]	2797±2017	**2216**	[1191-4279]	1409.2±1134	**1091**	[680-1780]
CYP2D6	183±120.7	**132**	[88.6-319.5]	**100±19**	**94**	[87-133]	70.8±50	**52.7**	[23-120]	32.9±16.3	**39.6**	[17-45.5]
CYP2E1	1250±452	**1209.5**	[857-1557]	**787.7±287**	**718**	[397-1207]	571.8±387.7	**471**	[384-778]	216.8±136.6	**165**	[124-283]
CYP2J2	23±19	**19**	[6.7-35]	**9.4±4**	**9**	[4.4-13.6]	6±4	**5.5**	[2.6-9.5]	2.9±2.4	**1.9**	[1-4.9]
CYP3A4	2719±2864	**1498**	[704-7597]	**917±637**	**891.6**	[89-1713]	558±460	**347**	[200-1129]	516.7±841	**306.5**	[200-493]
CYP3A5	974±983	**880**	[41-2001]	**565±613**	**464.7**	[36-1296]	53.4±25.8	**53.4**	[30.5-89.5]	529.5±470	**508.5**	[37.6-1063]
CYP3A7	ND	**ND**	ND	**139±56**	**142.6**	[8.8-193]	115.6±54.7	**92.3**	[72-224]	70.8±11.4	**68**	[56-87]
CYP4F2	357.5±195	**410**	[103-521]	**265±143**	**235**	[124-508]	136±70.7	**118**	[72-203]	76.6±37	**69**	[45.5-123]
UGT1A1	694.7±517	**589**	[166.5-1323]	**1183.7±534**	**1245**	[413-1800]	448±216.9	**407**	[239-630]	458±317.9	**360**	[156-701]
UGT1A3	110.7±87	**146.6**	[11.5-174]	**20.4±3**	**20.4**	[18-23]	52.7±23.6	**43.6**	[23.6-79]	33.6±21.5	**24.7**	[10-65.5]
UG**T1A4**	790±457	**697**	[532-928.4]	**383±33**	**383**	[350-416]	504.6±316.9	**460**	[208-1105]	192.4±98.7	**231.4**	[83.4-312.2]
UGT1A6	208±97	**186.7**	[145.5-233]	**136.7±56**	**139**	[70-215]	101.6±81.8	**81.5**	[50.5-122]	58.9±34	**43.5**	[40.7-73]
UGT1A9	689.7±425	**561**	[357-1209]	**648±263**	**760.5**	[173-855]	310.5±192.9	**267**	[132-443]	180.7±128	**157**	[89-240]
UGT2B15	725±335	**729.7**	[436-1045]	**347±94**	**315**	[273-454]	446±273	**392.7**	[205-611]	167.6±54.8	**169**	[69-233]
UGT2B4	1091.8±570	**945.5**	[619-1270]	**725±238**	**654**	[454-1059]	497.5±346	**382**	[307-466]	227.7±93	**233**	[145-272]
UGT2B7	3302.6±1470	**3122.5**	[2148-4993]	**1894.5±993**	**1715**	[863-3594]	1111±703	**927.5**	[540-1623]	464.7±180.6	**406**	[318-636]
UGT2B17	137.7±136	**81.6**	[11.7-419.4]	**ND**	**ND**	ND	48.4±NA	**48.4**	ND	ND	**ND**	ND
ATP1A1	105.8±41	**94**	[75-134]	**107.7±42**	**119**	[26.6-142]	64.8±29.4	**55**	[48-79.6]	50±18.8	**49.5**	[36-65.4]

CES1	10552±5590	**8790.8**	[6964-12270]	**4637.8±2190**	**4649**	[1280-7951]	3112.5±1576	**2431**	[1999-4533]	1145±704	**787**	[721-1655]
CES2	669±449	**513**	[384-1283]	**363±103**	**321**	[247-488]	252.5±102	**246**	[145-376]	126.5±41.5	**121.6**	[91.5-159]
EPHX1	11417.6±4814	**10269**	[8475-13749]	**6395.8±2429**	**6052**	[3682-9757]	4182.7±2068	**3453**	[2761-4815]	2002±1249	**1506**	[1261-2586]
FMO3	2600.5±1100	**2221.8**	[2003-3064]	**1323.7±305**	**1391**	[866-1668]	887±366.6	**964**	[489-1146]	469.4±298.4	**424.6**	[216.8-601]
FMO5	753±602	**492.5**	[317-1587]	**375.4±186**	**342**	[183-603]	323±245.8	**248**	[104-463]	129.6±105.8	**107.7**	[49-158]
MGST1	3922.6±1452	**3632.5**	[2596-4294]	**2960.4±1027**	**3054**	[1647-4503]	2143±1199	**2049**	[1235-2380]	1161.6±508.8	**1094**	[664-1558]
MGST3	292.8±115	**258**	[188.8-403]	**128.4±50**	**126**	[49.6-201.5]	120.5±59.5	**111.7**	[69-137]	81±31.8	**72**	[53.3-112]
POR	1119±477	**1051.8**	[775-1191]	**690.9±530**	**505.7**	[113-1610]	600±380	**553.8**	[256-1204]	481±457.9	**371**	[278-470.5]
P-gp, MD**R1**	9.4±3	**8.2**	[5.8-14]	**11.4±5**	**10.8**	[7.2-20]	5.6±2.4	**5**	[3.9-6]	4.5±1.8	**4.5**	[3-6.3]
BSEP	11.9±5	**11**	[8.6-14]	**6.7±3**	**6**	[3-12.5]	5±3.7	**4**	[2.8-5.4]	3.8±2.3	**3**	[2.1-5.5]
MDR3	7±4	**5.9**	[1.4-11.8]	**4.4±2**	**4.7**	[0.5-7]	3.4±3.7	**2.4**	[0.9-4.4]	2.8±2.9	**2**	[0.9-5.4]
MRP2	16±6	**14.7**	[12.2-17.3]	**7±3**	**6.7**	[2.7-11.5]	5±2.5	**5**	[3-8.7]	3.1±2	**2.7**	[2.1-4.3]
MRP3	10.5±6	**9**	[6-17]	**8.4±5**	**7.3**	[2.2-15]	5.4±2.7	**5**	[3-6]	5.6±2.7	**5**	[3.4-7.9]
MRP4	1±0.7	**1.1**	[0.03-2]	**0.6±0**	**0.4**	[0.2-1.2]	0.5±0.3	**0.7**	[0.01-1]	0.9±1.5	**0.3**	[0.05-0.7]
MRP6	19±8	**16.8**	[13.5-25]	**11.7±6**	**11**	[4-20.5]	7.5±7.7	**5**	[3-11]	4.3±1.9	**4**	[2.9-5.8]
BCRP	1.2±1	**0.4**	[0.14-2.7]	**0.4±0**	**0.3**	[0.1-0.9]	0.5±0.2	**0.5**	[0.2-0.6]	38.4±65.7	**0.4**	[0.38-0.49]
NTCP	71±17	**71**	[57.8-85.4]	**40.4±21**	**40**	[20.8-60.9]	33±19.7	**28.6**	[16.5-76.5]	16±7	**16.7**	[8.2-24.8]
ASBT	0.9±0.8	**0.4**	[0.17-2.2]	**0.4±0**	**0.2**	[0.1-1]	0.5±0.4	**0.3**	[0.15-1]	13.6±37.7	**0.2**	[0.08-0.6]
MCT-**1**	30±10	**32**	[20.5-37.8]	**27.7±12**	**31.7**	[9.4-42.8]	18.4±13	**15**	[10.5-18.3]	11±3	**11**	[8-13.4]
OCT-**1**	85±4	**86.7**	[80.8-88]	**41.7±12**	**39**	[31.2-62.7]	26.8±14.4	**20**	[16.3-40.8]	16.7±8.4	**16**	[7-32.8]
OCT-**3**	10.8±4	**9.4**	[6.2-15.9]	**9.8±5**	**8.9**	[3.1-18]	5.8±3.4	**4.6**	[4.2-7]	7±9	**3.4**	[2.6-8.3]
OAT2	29±14	**29.4**	[15.5-41.8]	**23±13**	**19**	[10.8-43.6]	12.4±7.7	**10**	[7.5-15.4]	7.7±2.7	**7.3**	[5.3-10.3]
OAT4	4±3	**3.4**	[2.2-5.1]	**1.8±1**	**1.7**	[0.7-2.7]	1.2±0.7	**1**	[0.6-2.8]	0.9±0.6	**0.8**	[0.37-1.8]
OATP1A2	3.3±3	**3**	[0.3-6.5]	**0.1±0**	**0.1**	[0.005-0.28]	0.7±0.6	**0.5**	[0.2-1.7]	1.3±2.6	**0.2**	[0.09-6.7]
OATP1B1	34±12	**33**	[23.2-41.5]	**16±4**	**15**	[11.6-22.7]	15.9±10.7	**13.8**	[8.2-16.8]	11.9±7	**9.8**	[6.4-28.3]
OATP1B3	31.8±14	**27**	[21-63.1]	**7±0**	**7**	ND	6±4.2	**5.6**	[2-10.5]	3.8±3.4	**2.4**	[1.4-7.7]
OATP2B1	48.5±17	**41**	[36.2-72]	**29.6±15**	**32**	[11.8-52.5]	18±11	**14.4**	[12.2-24.8]	12±5.5	**10.8**	[7-16.4]

CP, Child-Pugh score; 95% CI, 95% confidence interval around the median.

Table S9. Observed and simulated pharmacokinetic parameters in healthy and cirrhosis populations using either Simcyp V19 default settings or the change in the protein abundance from the current study.

Drug	AUC_{obs} in healthy ng.h/ml	AUC_{obs} in cirrhosis ng.h/ml	AUC_{pred} in healthy ng.h/ml	AUC_{pred} Proteomic in cirrhosis ng.h/ml	AUC_{pred} Simcyp in cirrhosis ng.h/ml	AUR_{obs}	$AUCR_{pred}$ Proteomics	$AUCR_{pred}$ Simcyp	$AUCR_{pred/obs}$ Proteomics	$AUCR_{pred/obs}$ Simcyp
**Repaglinide**	91.6±6.7	368.9±233.4	84.3±66	414±303	236±180	4.1	4.9	2.8	1.19	0.68
**Dabigatrane etexilate**	937±649	922±965	1014.3±649	879±523	885±526	0.98	0.87	0.87	0.89	0.89
**Zidovudine**	1388.4±374	CPA: 4714.6±1909<br>CPB: 4842.1±1289<br>CPC: 6321.4±1624	1412.4±635	CPA: 3026.4±1456<br>CPB: 4839.2±2123<br>CPC: 8078.1±3084.5	CPA: 1867.7±940<br>CPB: 2196.5±1088<br>CPC: 2515.7±1228	CPA:3.4<br>CPB: 3.5<br>CPC: 4.6	CPA:2.1<br>CPB:3.4<br>CPC:5.7	CPA:1.3<br>CPB:1.6<br>CPC:1.8	CPA:0.62<br>CPB:0.97<br>CPC:1.2	CPA:0.38<br>CPB:0.46<br>CPC:0.39

AUC, area under concentration-time profile; AUCR, ratio of AUC in cirrhosis population relative to healthy population; obs, observed data from clinical studies; pred, predicted by the model; Proteomics, predicted data by the model after applying the change in the abundance of protein in cirrhosis population relative to control; Simcyp, predicted data by the model after applying default abundance settings in the Simcyp simulator

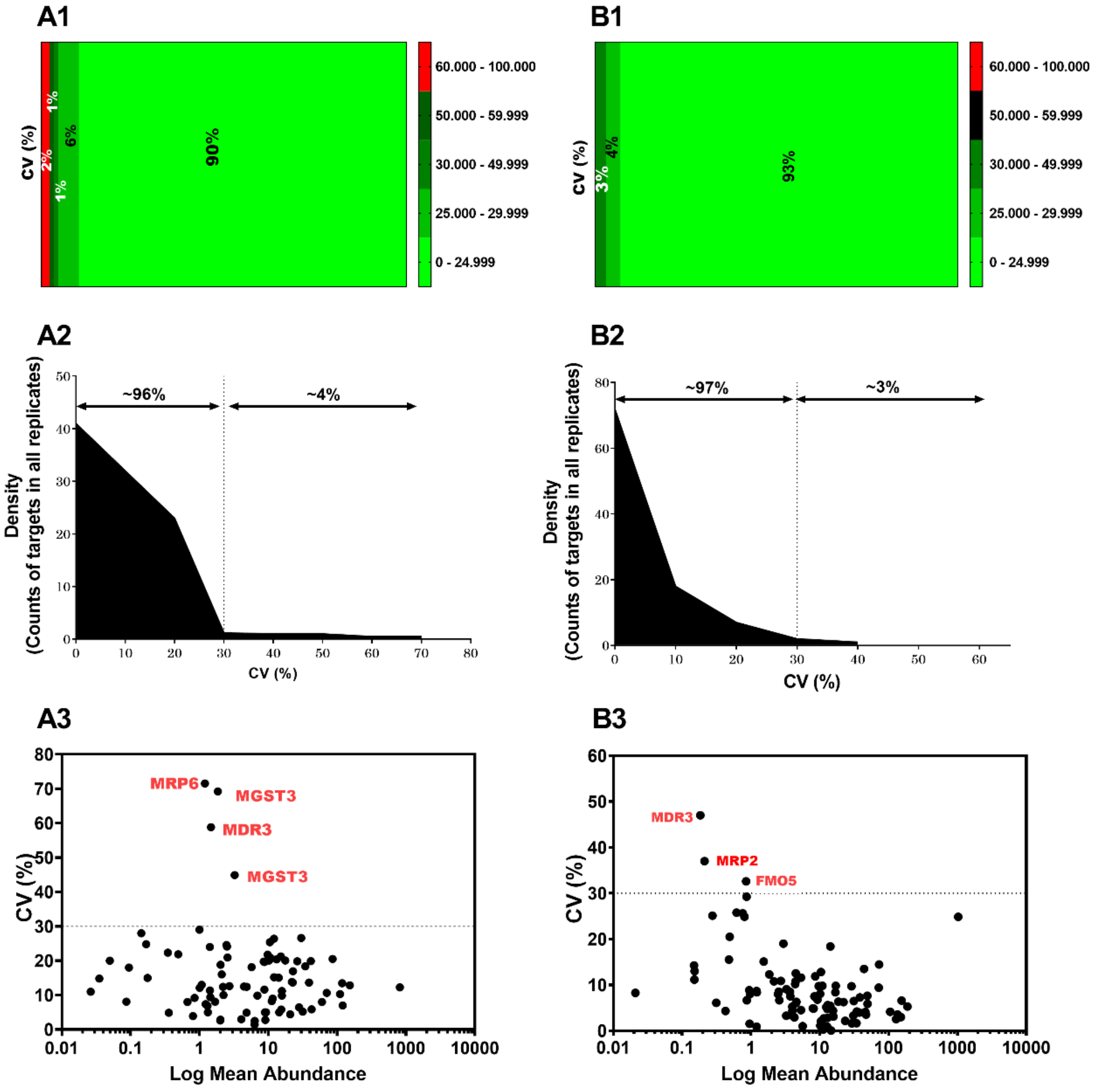

Figure S1. Technical (A1, 2, 3) and batch-to-batch (B1, 2, 3) variability represented by percent coefficients of variation (%CV) for all targets in a set of QC samples (n = 10) ;the density of targets at each %CV value (A2, B2), and %CV against log mean abundance of the proteins (A3, B3).

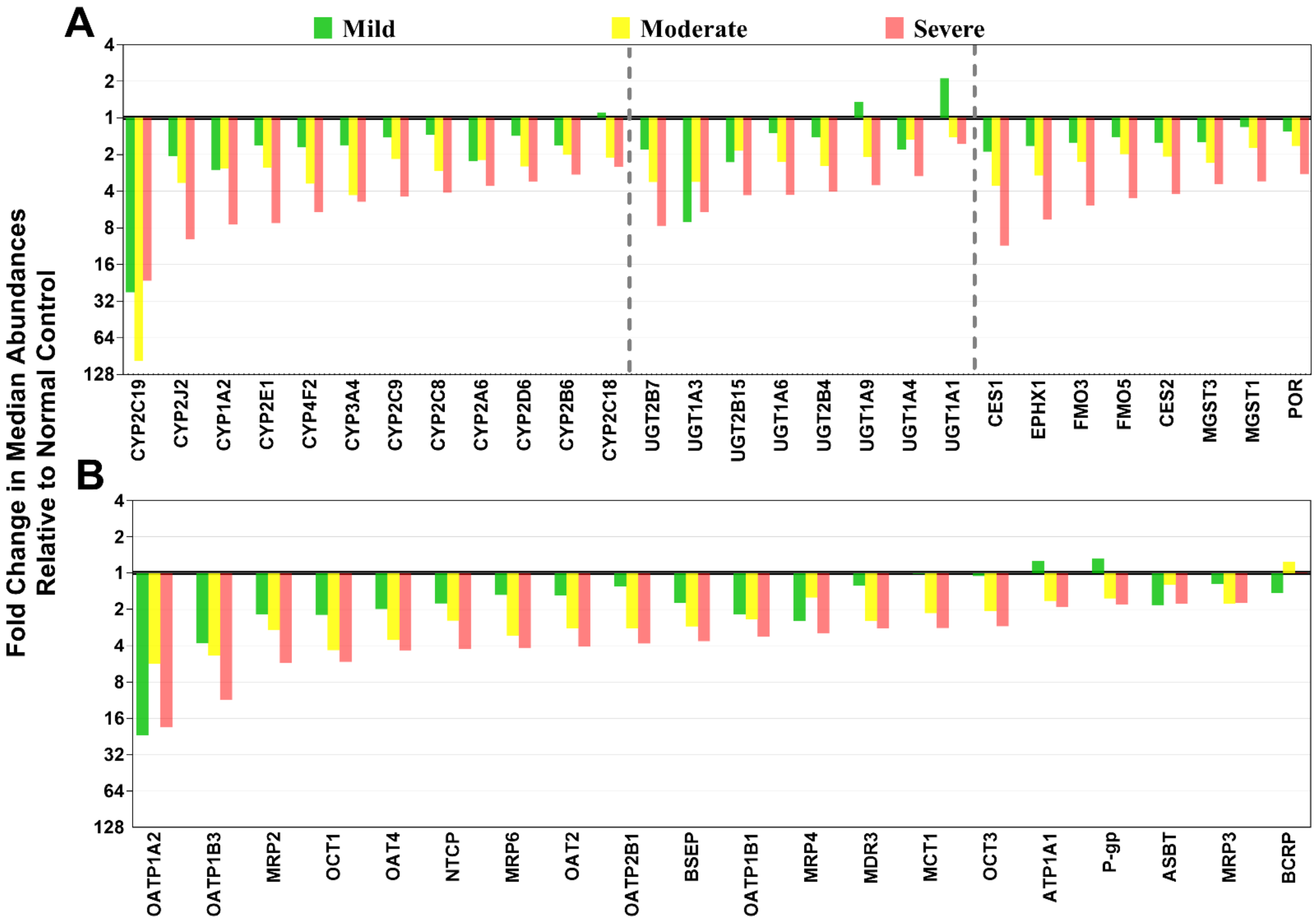

Figure S2. The fold change in the median abundances of different metabolizing enzymes (A) and transporters (B) with various levels of cirrhosis disease severity (mild, moderate, and severe). The blue and red arrows represent the direction of change relative to the control levels in histologically normal samples.

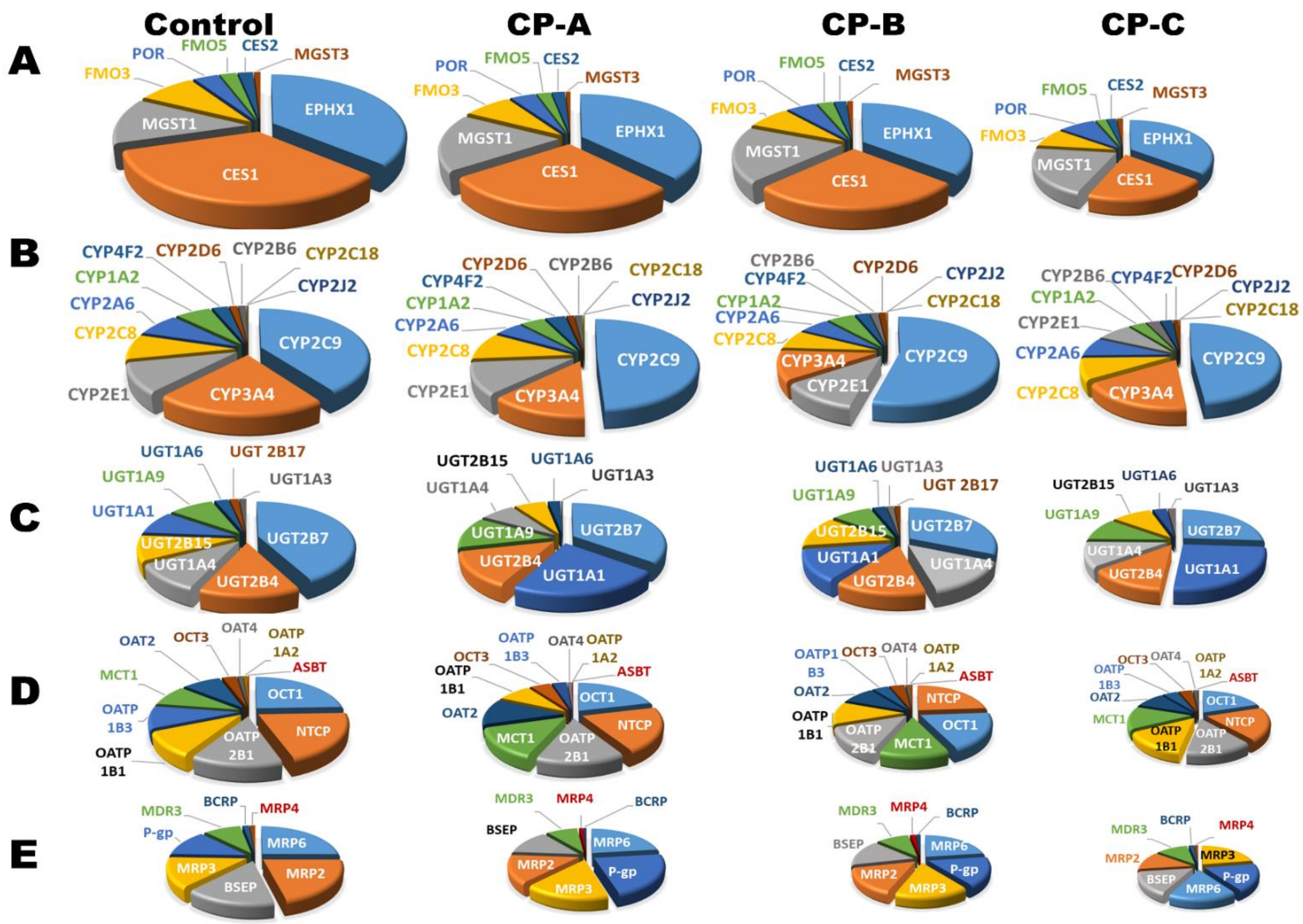

Figure S3. Pie charts representing relative abundance distribution of NonCYP, NonUGT enzymes (A), CYPs (B), uridine-5'-diphospho-glucuronosyltransferases (C), SLC transporters (D), and ABC transporters (E) per gram of liver tissue from the normal control, mild (CP-A), moderate (CP-B), and severe (CP-C) cirrhosis groups. The pie sizes are proportional to the Log transformed sum of the abundances of the contributing proteins.

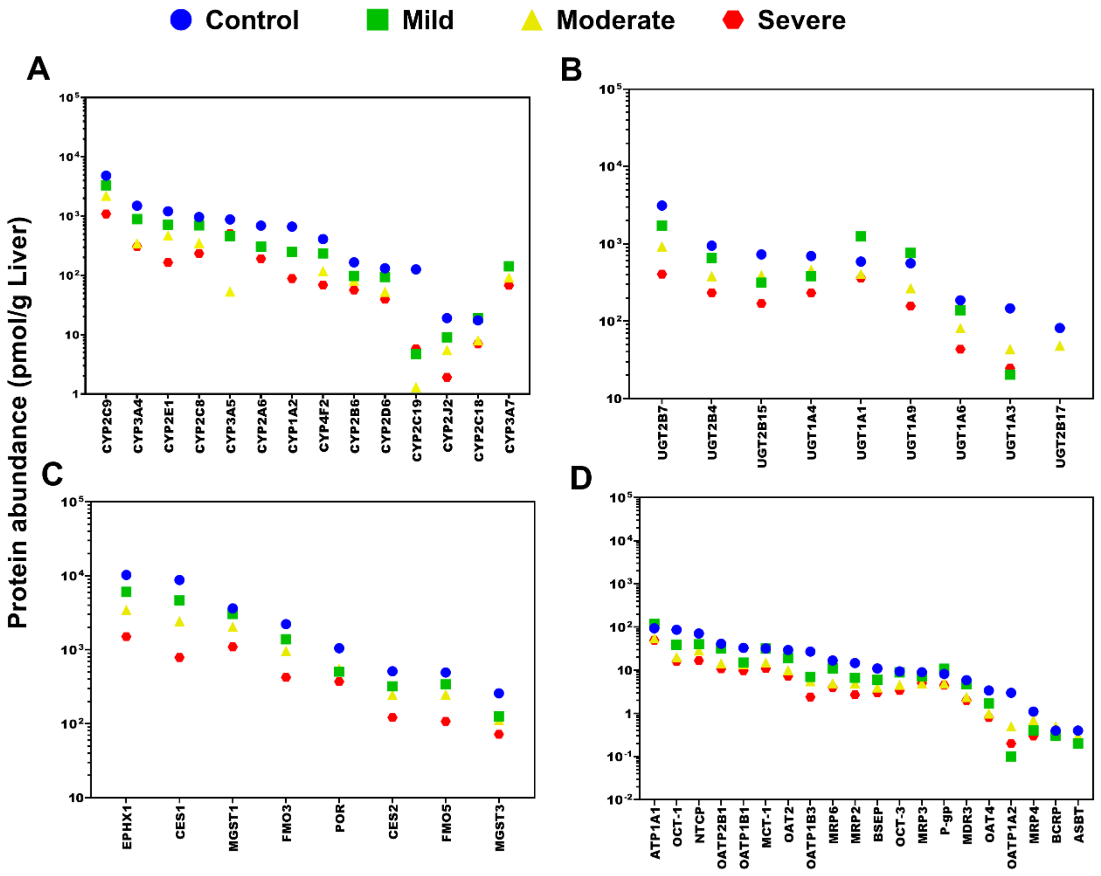

Figure S4. Median abundance values of each target protein (A: CYPs, B: UGTs, C: Non-CYP non UGT microsomal enzymes, D: Transporters) representing the 4 group of samples (control livers, mild, moderate, and severe cirrhosis livers).

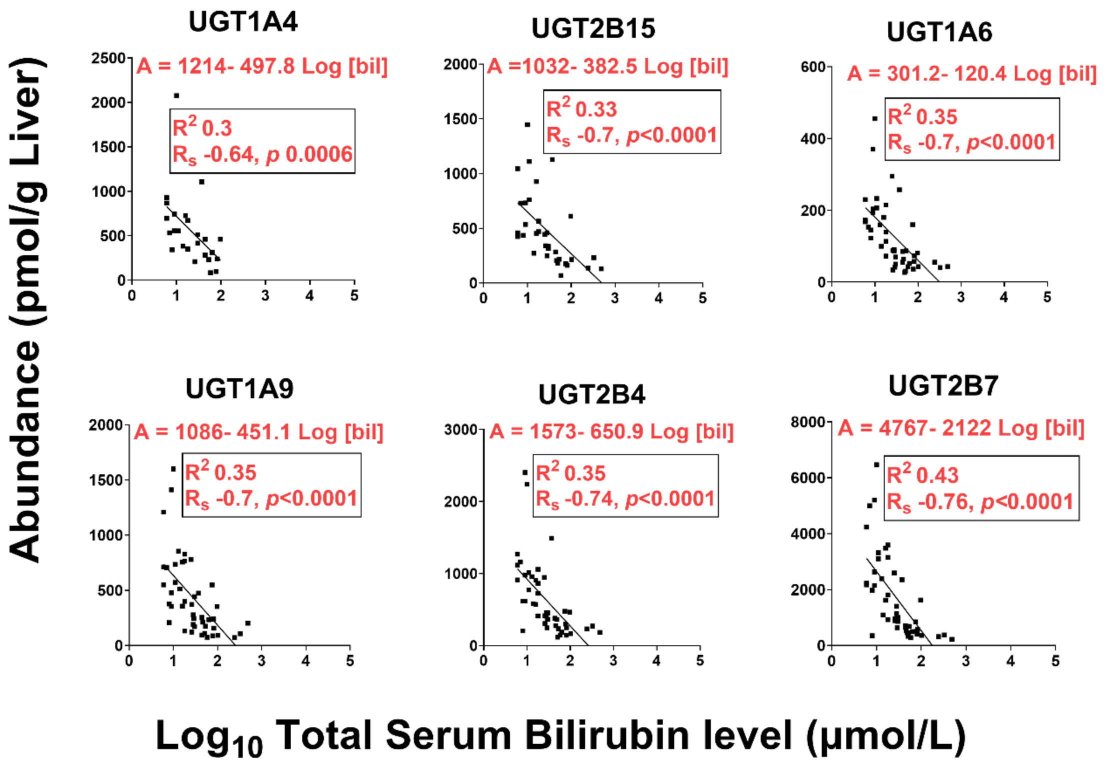

Figure S5. Correlations between abundance of liver uridine-5'-diphospho-glucuronosyltransferases (in pmol/g tissue), and log-transformed total serum bilirubin levels; Log [bil] (μmol/L) in liver donors assessed by Spearman correlation test (R_s) and linear regression (R^2).

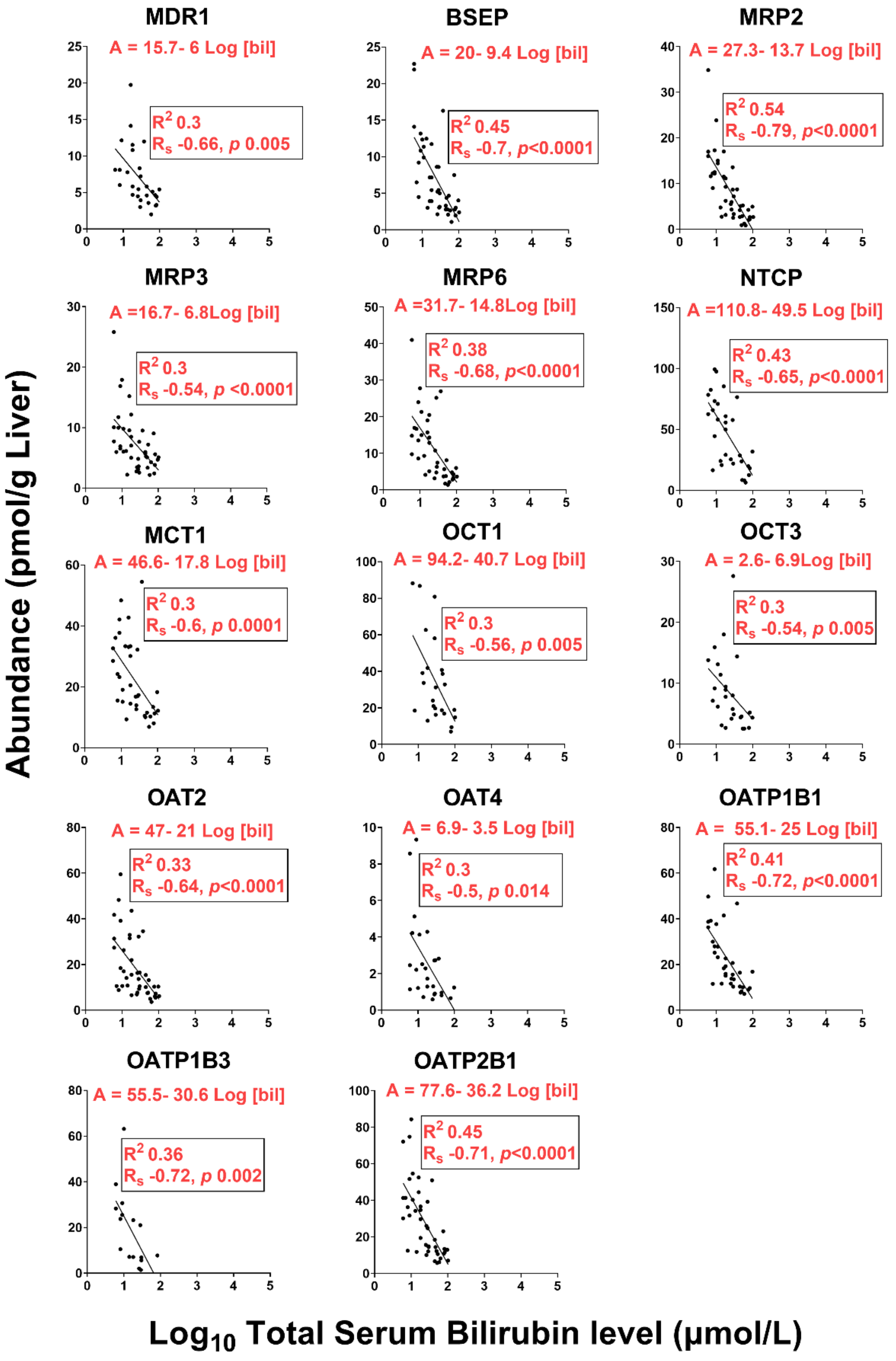

Figure S6. Correlations between abundance of liver transporters (in pmol/g tissue), and log-transformed total serum bilirubin levels; Log [bil] (µmol/L) in liver donors assessed by Spearman correlation test (R_s) and linear regression (R^2).

References

(1) Hatorp, V.; Walther, K. H.; Christensen, M. S.; Haug-Pihale, G. Single-Dose Pharmacokinetics of Repaglinide in Subjects with Chronic Liver Disease. *J. Clin. Pharmacol.* **2000**, *40* (2), 142–152. https://doi.org/10.1177/00912700022008793.

(2) Stangier, J.; Stähle, H.; Rathgen, K.; Roth, W.; Shakeri-Nejad, K. Pharmacokinetics and Pharmacodynamics of Dabigatran Etexilate, an Oral Direct Thrombin Inhibitor, Are Not Affected by Moderate Hepatic Impairment. *J. Clin. Pharmacol.* **2008**, *48* (12), 1411–1419. https://doi.org/10.1177/0091270008324179.

(3) Taburet, A.-M.; Naveau, S.; Zorza, G.; Colin, J.-N.; Delfraissy, J.-F.; Chaput, J.-C.; Singlas, E. Pharmacokinetics of Zidovudine in Patients with Liver Cirrhosis. *Clin. Pharmacol. Ther.* **1990**, *47* (6), 731–739. https://doi.org/10.1038/clpt.1990.101.
